# Monoacylglycerol Lipase Inhibitor JZL184 Improves Behavior and Neural Properties in Ts65Dn Mice, a Model of Down Syndrome

**DOI:** 10.1371/journal.pone.0114521

**Published:** 2014-12-04

**Authors:** Larisa V. Lysenko, Jeesun Kim, Cassandra Henry, Anna Tyrtyshnaia, Rebecca A. Kohnz, Francisco Madamba, Gabriel M. Simon, Natalia E. Kleschevnikova, Daniel K. Nomura, R . Alan B. Ezekowitz, Alexander M. Kleschevnikov

**Affiliations:** 1 Department of Neurosciences, University of California San Diego, La Jolla, CA, United States of America; 2 Abide Therapeutics, Inc., San Diego, CA, United States of America; 3 School of Biomedicine, Far Eastern Federal University, Sukhanova 8, Vladivostok, Russian Federation; 4 Department of Nutritional Sciences and Toxicology, University of California, Berkeley, California, United States of America; Cleveland Clnic Foundation, United States of America

## Abstract

Genetic alterations or pharmacological treatments affecting endocannabinoid signaling have profound effects on synaptic and neuronal properties and, under certain conditions, may improve higher brain functions. Down syndrome (DS), a developmental disorder caused by triplication of chromosome 21, is characterized by deficient cognition and inevitable development of the Alzheimer disease (AD) type pathology during aging. Here we used JZL184, a selective inhibitor of monoacylglycerol lipase (MAGL), to examine the effects of chronic MAGL inhibition on the behavioral, biochemical, and synaptic properties of aged Ts65Dn mice, a genetic model of DS. In both Ts65Dn mice and their normosomic (2N) controls, JZL184-treatment increased brain levels of 2-arachidonoylglycerol (2-AG) and decreased levels of its metabolites such as arachidonic acid, prostaglandins PGD2, PGE2, PGFα, and PGJ2. Enhanced spontaneous locomotor activity of Ts65Dn mice was reduced by the JZL184-treatement to the levels observed in 2N animals. Deficient long-term memory was also improved, while short-term and working types of memory were unaffected. Furthermore, reduced hippocampal long-term potentiation (LTP) was increased in the JZL184-treated Ts65Dn mice to the levels observed in 2N mice. Interestingly, changes in synaptic plasticity and behavior were not observed in the JZL184-treated 2N mice suggesting that the treatment specifically attenuated the defects in the trisomic animals. The JZL184-treatment also reduced the levels of Aβ40 and Aβ42, but had no effect on the levels of full length APP and BACE1 in both Ts65Dn and 2N mice. These data show that chronic MAGL inhibition improves the behavior and brain functions in a DS model suggesting that pharmacological targeting of MAGL may be considered as a perspective new approach for improving cognition in DS.

## Introduction

Genetic alterations or pharmacological treatments affecting brain levels of endocannabinoids have profound effects on synaptic and neuronal properties and, under certain some conditions, may improve higher brain functions. The most abundant endocannabinoid in the brain is 2-arachidonoylglycerol (2-AG). Similar to other lipid signaling molecules, levels of 2-AG are controlled by a balance of biosynthesis and degradation [1]. The principal hydrolytic enzyme responsible for the degradation of 2-AG is monoacylglycerol lipase (MAGL) [2], [3]. Consequently, genetic or pharmacological suppression of MAGL activity results in a robust increase of the brain levels of 2-AG and a concomitant reduction of arachidonic acid and downstream eicosanoid metabolites [4], [5], [6]. Thus, inhibition of MAGL may simultaneously increase levels of 2-AG, resulting in activation of cannabinoid receptors, and reduce the release of eicosanoids, resulting in suppression of pro-inflammatory signaling in the nervous system. Recently, it was shown that inhibition of MAGL with JZL184, the most selective and potent MAGL inhibitor [4], improved synaptic plasticity and memory in a mouse model of Alzheimer's disease (AD) [7]. Furthermore, MAGL KO mice also exhibited increased synaptic plasticity and memory [8], suggesting that disruption of MAGL activity could positively affect higher brain functions. Finally, genetic [9] or pharmacological [7] inactivation of MAGL robustly suppressed accumulation of β-amyloid (Aβ) in a mouse AD model.

Down syndrome (DS) is a developmental disorder caused by triplication of chromosome 21 [10]. Mouse genetic models of DS carry an extra copy of genes homologous to those on human chromosome 21. One of the most widely used genetic models of DS, segmentally trisomic Ts65Dn mice, have three copies of most of the genes on mouse Chr 16 that are homologues of human Chr 21 genes, including the *App* gene. Ts65Dn mice exhibit abnormalities in brain structure, cognition, and behavior similar to those observed in people with DS [11], [12], [13], [14], [15], [16], [17]. Thus, both people with DS and Ts65Dn mice have deficient hippocampus-dependent memory [18], [19], [20], [21], [22], working memory [23], [24], [25], exhibit multiple dendritic, synaptic, and neuronal abnormalities [26], [27], [28], [29], and show the AD type pathology later in life [30], [31], [32].

Here we examined the effects of JZL184 on the neural properties and behavior of aged Ts65Dn mice. We observed that chronic suppression of MAGL increased brain levels of 2-AG, restored spontaneous locomotor activity, and improved long-term memory and synaptic plasticity in Ts65Dn mice. In addition, JZL184-treatment decreased levels of Aβ40 and Aβ42 in both Ts65Dn and 2N mice. These results point to MAGL as a novel prospective therapeutic target for improving cognition and, possibly, ameliorating AD-type neuropathology during aging in individuals with DS.

## Materials and Methods

### Animals

Segmental trisomy 16 (Ts65Dn) mice were purchased from the Jackson Laboratory, Bar Harbor, ME, stock #001924. Diploid (2N) littermate mice served as control. Mice were males and housed 2 to 4 per cage with a 12 h light-dark cycle and ad lib access to food and water. Genotype of all animals was confirmed after completing experiments. For genotyping, tail samples were used to extract genomic DNA. A quantitative polymerase chain reaction protocol developed by the Jackson Laboratory, Bar Harbor, ME (http://www.jax.org/cyto/quanpcr.html) was used to measure expression of the Mx1 gene, which is present in three copies in Ts65Dn. In addition, all mice were prescreened for *Pde6brd1* homozygosity, a recessive retinal degeneration mutation that results in blindness [33], and only animals free of retinal degeneration were used.

The experiments were conducted in accordance with the National Institutes of Health guidelines and with an approved protocol from the University of California San Diego (UCSD) Institutional Animal Care and Use Committee.

### Experimental design and treatments

Two cohorts of male mice, with the average age of 11.4±0.2 mo (major cohort, n = 78) and 9.5±0.1 mo (additional cohort, n = 26) at the beginning of experiments were used. The mice were handled for 5 min once a day for 7 consecutive days before the experiments began. Body weight was measured daily before treatment. JZL184 or vehicle was injected intraperitoneally (i.p.) once a day for 4 weeks at a dose of 8 mg/kg (major cohort) or 40 mg/kg (additional cohort), at a volume of 10 µL/g of body mass. The treatment doses were selected on the basis of previously published data showing that JZL184 selectively inhibits MAGL at the dose of 8 mg/kg, and inhibits both MAGL and fatty acid amid hydrolase (FAAH) at the dose of 40 mg/kg [4]. Thus, comparison between the results for the two doses could allow for the differentiation between the effects of suppressing MAGL alone or both MAGL and FAAH together.

The animals were split into 4 groups (2N Veh, 2N JZL, Ts Veh, and Ts JZL) and examined in behavioral tests during weeks 3–4 of injections. The injections were performed 1–2 h before the tests during these experiments. The major cohort of mice was split into 4 groups as follows: 2N Veh - 22, 2N JZL - 20, Ts Veh - 18, and Ts JZL - 18. After completing the behavioral tests, these animals were used in studies of synaptic plasticity (2N Veh - 6, 2N JZL - 4, Ts Veh - 4, and Ts JZL - 4), Western blot (2N Veh - 6, 2N JZL - 5, Ts Veh - 6, and Ts JZL - 5), and lipidomic analysis (2N Veh - 8, 2N JZL - 8, Ts Veh - 7, and Ts JZL - 7). In addition, the second half of the animal's brain used for lipidomic analysis was also used for measuring levels of beta amyloid. Several mice died during or after the behavioral tests (2N Veh - 2, 2N JZL - 3, Ts Veh - 1, and Ts JZL - 2). The additional cohort of mice was split in the following groups: 2N Veh - 8, 2N JZL - 7, Ts Veh - 5, and Ts JZL - 5. These animals were tested only for locomotor activity, and then some of the animals were used for lipidomic analysis (2N Veh - 4, 2N JZL - 4, Ts Veh - 4, and Ts JZL - 4).

### Behavioral testing

Behavioral studies were performed during the light cycle between 7:00 a.m. and 7:00 p.m. To minimize olfactory cues from previous trials, each apparatus was thoroughly cleaned with 10% ethanol after each animal. On the day of testing, mice were left in their home cages in the room used for the experiment at least 1 hour prior to the onset of the study for habituation. All behavioral tests and procedures were performed by personnel blind to both genotype and treatment group.

#### Spontaneous locomotor activity

Spontaneous locomotor activity was evaluated in square activity chambers (43.2×43.2×20 cm) made of acrylic glass and equipped with three planes of infrared detectors (Med Associates Inc, St. Albans, VT). Four mice were tested concurrently in individual chambers. The area of a chamber was divided virtually on center (20 cm×20 cm, zone 1) and periphery (the rest of the chamber, zone 2). The activity chambers were located within sound-attenuating boxes (66×55.9×55.9 cm) with a built-in internal fan for background noise (65 dB) and lighting for ambient illumination. For testing, an animal was placed in the center of the testing arena under bright ambient light and allowed to move freely for 10 minutes. The movements were monitored by an automated tracking system (Med Associates Activity Monitor, version 5.93.773).

#### Y-maze

Y-maze testing was performed using an apparatus with three equal arms (30 cm length, 10 cm width, and 20 cm height), made of opaque acrylic glass. A mouse was placed at the maze center and was allowed 5-min of exploration of the environment. An arm entry was scored when the mouse entered the arm with all four paws. Total number of entries (N) and number of ‘correct’ triplets (M, consecutive choices of each of the three arms without re-entries) was evaluated. The alternation rate was computed according to the formula: R (%) = M*100/(N-2).

#### Novel place and object recognition tests

Novel place recognition (NPR) and novel object recognition (NOR) were tested using the Bevins and Besheer protocol [34]. Mice were habituated in a black rectangular chamber made of acrylic glass (31×24 cm, height 27 cm) for 10 min on 2 consecutive days under dim ambient lighting conditions. The activity of mice was recorded with a video camera. Each test had two phases – acquisition and testing. For acquisition, two identical objects were placed in diagonally opposite corners of the chamber, 8–9 cm from the walls. A mouse was placed at the mid-point between the objects. After allowing for 10 min exploring the objects, the mouse was returned to the colony. The testing was performed 10 min (NPR) or 24 hours (NOR) after the acquisition. In the NPR tests, one of the objects was moved in a new location in another corner of the chamber, while the other object remained at its former spatial location. In the NOR tests, one of the objects was replaced with a novel object of the same height and volume, but different shape and appearance. For testing, the mouse was again placed in the chamber to explore the objects for 3 min. The amount of time spent exploring each object (nose sniffing and head orientation within <1.0 cm) was recorded and evaluated by operators blinded to genotype and treatment. The discrimination index was computed as R = Tnew*100/(Tnew+Told), were Tnew is the time spent exploring the new or relocated object, and Told is the time spent exploring the familiar or unmoved object.

### Electrophysiology

Transverse hippocampal slices were prepared as previously described [19], [35]. Mice were anesthetized with isoflurane before decapitation. The brain was quickly removed and immersed for 2 min in ice-cold artificial cerebrospinal fluid (ACSF) containing 119 mM NaCl, 2.5 mM KCl, 2.5 mM CaCl2, 1.3 mM MgSO4, 1 mM NaH2PO4, 26 mM NaHCO3, 10 mM glucose, osmolarity 305 mOsm, continuously bubbled with 95% O2-5% CO2, pH 7.4. The hippocampus was extracted and cut in ice-cold ACSF with a vibratome (Leica VT1000S) into 350-µm-thick slices, which were allowed to recover in oxygenated ACSF at room temperature for at least 2 h prior to experimental recordings.

A slice was transferred into the submerged recording chamber and superfused with ACSF at a constant rate of 2.5 ml/min at 32°C. Recording electrodes were made of borosilicate glass capillaries (1B150F, World Precision Instruments, Sarasota, FL) and filled with ACSF (resistance ∼0.5 MΩ). Monopolar stimulating electrodes were made of Pt/Ir wires of diameter 25.4 µm (PTT0110, World Precision Instruments, Sarasota, FL) and had 100-µm-long exposed tips. Both the stimulating and recording electrodes were inserted under visual control perpendicular to the slice surface into the CA1 *stratum radiatum* at a distance 250–300 µm from each other. The initial slope of the field excitatory postsynaptic potentials (fEPSP) was measured at latencies 0.1–0.9 ms. Testing stimuli (duration 100 µs, current 70 µA) evoked field responses with amplitudes of 70–80% of maximum. LTP was induced by theta-patterned tetanizations consisting of 4 trains of stimuli, 4 pulses at 100 Hz each, applied with an inter-train interval of 200 ms (5 Hz).

### Lipidomic analysis

Lipid species were analyzed by targeted single reaction monitoring (SRM)-based methods on an 6430 Agilent QQQ-LC/MS using previously described procedures [5], [36]. Briefly, mice were sacrificed by cervical dislocation and their brains were rapidly removed and flash frozen. Brains were dounce homogenized in 6 ml 1∶1 ethyl acetate: hexane and 2 ml phosphate buffered saline with the inclusion of internal standards (10 nmol dodecylglycerol, 10 nmol pentadecanoid acid, 1 nmol d9-PGE2, 1 nmol d5-2-AG, 1 nmol d4-AEA). The resulting mixture was centrifuged at 1300 rpm and the organic layer was extracted, dried under N2, and the dried lipid extract was resuspended in 150 µL chloroform. An aliquot was injected onto LC/MS. Lipid metabolites were separated on a reverse phase C5 column (50 mm×4.6 mm with 5 micrometer diameter particles). For LC separation, mobile phase A consisted of 95∶5 water: methanol and mobile phase B consisted of 60∶35∶5 isopropanol:methanol:water. Formic acid (0.1%) and 5 mM ammonium formate or ammonium hydroxide (0.1%) was included to assist in ion formation in positive and negative ionization modes, respectively. SRM parameters are described previously [5], [36]. Endocannabinoid and eicosanoid metabolites were quantified based on integration of the area under the peak and normalization to appropriate internal standards. Other lipid metabolites were normalized to internal standards and relative values are reported.

### Western blot

The hippocampi were dissected on an ice-cold preparation table and homogenized in RIPA buffer (50 mM Tris-HCl, 1% NP-40, 0.25% Na-deoxycholate, 150 mM NaCl, 1 mM EDTA, 1 mM PMSF, 1 mM Na3VO4, 1 mM NaF) with 1 mg/mL protease inhibitor cocktail (aprotinin, leupeptin, pepstatin). The protein concentrations were determined using BCA protein Assay kit (Pierce, Rockford, IL). β–Actin was used as a reference protein. 20 µL (1 mg/mL) of total protein per lane were loaded onto precast 4–12% Bis-Tris gels (Invitrogen, Carlsbad, CA), and separated by electrophoresis at 90 volts for ∼2 hours. Proteins were transferred to a low-fluorescent, hydrophobic polyvinylidene difluoride (PVDF) membrane, with 0.45 µm pore size (Millipore, Billerica, MA; Cat# IPVH00010), and the membranes were blocked with 4% nonfat milk in TBS-T solution (20 mM Tris-HCl, 150 mM NaCl, 0.1% Tween-20, pH 7.6). Membranes were then incubated with following primary antibodies: rabbit polyclonal anti-BACE1 B690 C-Terminal (1∶1000, Covance Inc; Cat # PRB-617C, RRID: AB_10063987), rabbit polyclonal anti-App (1∶1000), mouse monoclonal anti-β-actin (1∶2000, Sigma-Aldrich, Cat# A5441, RRID: AB_476744). The blots were washed in TBS-T (3 times×10 min) followed by incubation with secondary antibodies: goat anti-rabbit IgG-HRP conjugates (1∶10’000, Jackson Immunoresearch Laboratories Inc; Cat# 111-035-144, RRID: AB_2307391) or goat anti-mouse (1∶10’000, Jackson Immunoresearch Laboratories Inc; Cat# 115-035-146, RRID:AB_2307392). The blots were washed in TBS-T and then processed with a developing system (Bio-Rad, Hercules, CA; Cat# 170-5061). Immunoblots were scanned with imaging system (Moleculer Imager ChemiDoc XRS+, Bio-Rad, USA) and the images were analyzed with ImageJ (NIH, USA).

### Aβ measurements

Brains of the experimental mice were extracted and flash-frozen. Hemi brain samples were homogenized and processed on ice with 250 µl/sample of 1X RIPA buffer (#9806, Cell Signaling Technology), with the addition of protease inhibitors (Complete, Roche), PMSF, and Na3VO4. Samples were centrifuged, supernatants collected, and stored in aliquots at −80°C. Protein levels for stock samples were determined using a BCA Protein Assay and samples were diluted to 4 mg/mL for the electro-chemi-luminescence assay. MSD electro-chemi-luminescence multiplex assay (Meso Scale Discovery, Gaithersburg, MD) was performed according to the manufacturer's specifications using mouse specific Aβ Peptide Panel 1 (4G8) V-PLEX Kit (Meso Scale Discovery, Cat. # K15199E-1, Lot K0040192). In short, a 96 well plate pre-coated with anti- Aβ40 and Aβ42 antibodies was incubated at room temperature for 1 h with Diluent 35, a 1% blocker solution. After the plate was washed 3 times with 1X MSD Wash Buffer, 25 µl of SULFO-TAG detection antibody solution was added to each well, followed by addition of 25 µl of calibrators or diluted samples (‘simultaneous incubation’ experimental format) and incubated for 2 h. After the incubation, the plate was washed 3 times with the wash buffer, 150 µl of 2X MSD Read Buffer T was added to the plate, and read immediately on the MSD Sector Imager 2400 (620 nm). Each sample was analyzed in duplicates and then averaged for a mean value. The data was analyzed using MSD workbench software.

### Chemicals

JZL184 was a gift from Abibe Therapeutics (La Jolla, CA). All other chemicals were purchased from Sigma–Aldrich (St. Louis, MO).

### Statistics

Data are given as means ± SEM. Repeated measurements two-way ANOVA with main effects of genotype and treatment, as well as Student's T-test were used to identify significant differences, and p<0.05 was taken as statistically significant. All tests were two-tailed.

## Results

Ts65Dn mice and their littermate 2N controls were i.p. injected once a day for 4 weeks with either the selective MAGL inhibitor JZL184 or vehicle. For the major cohort, a drug dose of 8 mg/kg was used. From previously published data, we expected that this dose of JZL184 would selectively inhibit MAGL and would not affect the activity of fatty acid amid hydrolase (FAAH), the primary enzyme hydrolyzing endocannabinoid anandamide (AEA) [37]. Behavioral tests were performed during weeks 3 and 4 of the treatment. Following these tests, hippocampal synaptic plasticity was examined, lipidomic analysis was performed, and brain levels of β-amyloid were measured. In addition, a smaller cohort of mice was injected with JZL184 at a dose of 40 mg/kg, which presumably suppressed the activity of both MAGL and FAAH [37]. Animals from this cohort were only used for studies of locomotor activity and for lipidomic analysis. The animals' body weight was monitored daily before the injections. At baseline, the body weight was significantly smaller in Ts65Dn vs. 2N mice (2N: 44.79±0.77 g, n = 40; Ts65Dn: 32.32±1.40, n = 31; p<0.0001). Measurements made on the last day of the treatment showed that the body weight was not affected by the JZL184 dose of 8 mg/kg (p = 0.31 for 2N JZL vs. 2N Veh; p = 0.71 for Ts JZL vs. Ts Veh), but it was significantly reduced by the dose of 40 mg/kg (p = 0.037 for 2N JZL vs. 2N Veh; p = 0.031 for Ts JZL vs. Ts Veh). Expressed in percentage points of the pre-injection values, the reduction in body weight was equal in the JZL184-injected Ts65Dn vs. 2N mice (2N: 6.8±2.6%; Ts65Dn: 6.4±1.1%, n.s.).

### Lipidomic analysis: Endocannabinoids and their metabolites

Levels of endocannabinoids, their downstream products, and other lipids were measured as was previously described [5]. In the vehicle-treated groups, baseline levels of 2-AG were increased in Ts65Dn vs. 2N mice by 1.48-fold (Table 1). In contrast, levels of downstream products of 2-AG, such as arachidonic acid (AA), prostaglandins PGD2, PGE2, PGFα, and PGJ2, were in general lower in Ts65Dn vs. 2N mice, but the differences were not significant. Likewise, there was no difference in the levels of AEA in Ts65Dn vs. 2N mice (Table 1).

**Table 1:** Effect of chronic JZL184 treatment on the brain levels of endocannabinoids and their metabolites.

	2N			Ts65Dn		
	Veh	JZL184	JZL184	Veh	JZL184	JZL184
		8 mg/kg	40 mg/kg		8 mg/kg	40 mg/kg
**2-AG**	100±8.8	392.9±47.4^ a^	547.8±50.1^a^	148.0±15.8^ a^	451.0±47.7^ a,b^	572.0±23.1^ a,b^
**AEA**	100±11.9	112.2±19.2	270.9±38.5^ a^	115.7±12.9	133.5±21.1	248.9±24.8^ a,b^
**AA**	100±9.1	30.3±2.3^ a^	20.1±1.7^ a^	91.4±4.1	27.7±3.0^ a,b^	23.9±1.2^ a,b^
**PGD2**	100±13.0	22.9±3.0	8.1±3.4^ a^	89.3±4.9	17.6±2.0^ a,b^	8.1±1.4^ a,b^
**PGE2**	100±14.1	29.0±4.4^ a^	16.1±3.9^ a^	85.3±8.9	34.3±5.0^ a,b^	25.0±4.4^ a,b^
**PGFα**	100±13.9	26.8±4.0^ a^	-	84.7±8.5	36.3±7.4^ a,b^	-
**PGJ2**	100±13.7	33.6±5.4^ a^	-	89.3±6.1	32.4±3.6^ a,b^	-

The values are given in percents of the ‘2N Veh’ group.

a) p<0.001. Significant difference vs. ‘2N Veh’ group.

b) p<0.001. Significant difference vs. ‘Ts65Dn Veh’ group.

Suppressing key enzymes involved in endocannabinoid hydrolysis would expectedly increase the levels of these endocannabinoids and reduce the levels of their downstream metabolites. In agreement with this expectation, JZL184 treatment considerably increased levels of 2-AG (F_1,26_ = 83.2; p = 1.39E-09) and reduced levels of AA (F_1,26_ = 147.3; p = 3.26E-12), as wells as several prostaglandins in both Ts65Dn and 2N mice (Table 1, Table S1). Thus, levels of 2-AG were increased in the JZL184-treated vs. vehicle-treated animals by several folds (2N: 8 mg/kg = 3.9-fold, 40 mg/kg = 5.5 fold; Ts65Dn: 8 mg/kg = 3-fold, 40 mg/kg = 3.8-fold). Two-way ANOVA showed no significant interaction between genotype and treatment (F_1,26_ = 0.02; p = 0.88) suggesting that these changes were similar in the Ts65Dn and 2N mice. In contrast, levels of AA and prostaglandins were reduced by the treatment to 18-36% of the baseline values (Table 1, Table S1). These changes were also statistically similar in Ts65Dn vs. 2N mice (p = 0.12-0.91) (Table 1, Table S1). Levels of AEA were not affected by the JZL184 dose of 8 mg/kg (F_1,26_ = 3.26; p = 0.08) but significantly increased by the JZL184 dose of 40 mg/kg (F_1,26_ = 37.33, p = 4.61E-12; 2N: 2.7-fold increase; Ts65Dn: 2.5-fold increase). These results suggest that, in congruence with previous findings [37], JZL184 selectively suppressed MAGL at the dose of 8 mg/kg, and suppressed both MAGL and FAAH at the dose of 40 mg/kg. All changes were similar in Ts65Dn vs. 2N mice.

The levels of several other lipids were also altered in Ts65Dn vs. 2N mice (Table S1). Interestingly, JZL184-treatment was able to normalize some of those changes. Thus, levels of alkyl PG C16∶0/20∶4, PG C16∶0/C18∶1, PG C18∶0/C18∶1, PG C18∶0/C20∶4, and TAG C16∶0/C20∶4/C16∶0 were reduced in the vehicle-treated Ts65Dn vs. 2N mice, and were increased to normal by the treatment. On the contrary, levels of alkyl LPG C16∶0 and LPG C20∶4 were increased in the vehicle-treated Ts65Dn vs. 2N group, and reduced to normal by the treatment. The meaning and importance of these changes is yet to be determined.

### Behavioral characterization

Behavioral tests were performed during weeks 3 and 4 of the treatment and followed the order: (i) Activity box (spontaneous locomotor activity and thigmotactic behavior); (ii) Y-maze (working memory and locomotor activity); (iii) Novel place recognition with a retention period of 10 min (short-term memory); (iv) Novel object recognition with a retention period of 24 h (long-term memory).

#### Spontaneous locomotor activity and thigmotactic behavior

**Figure 1:**
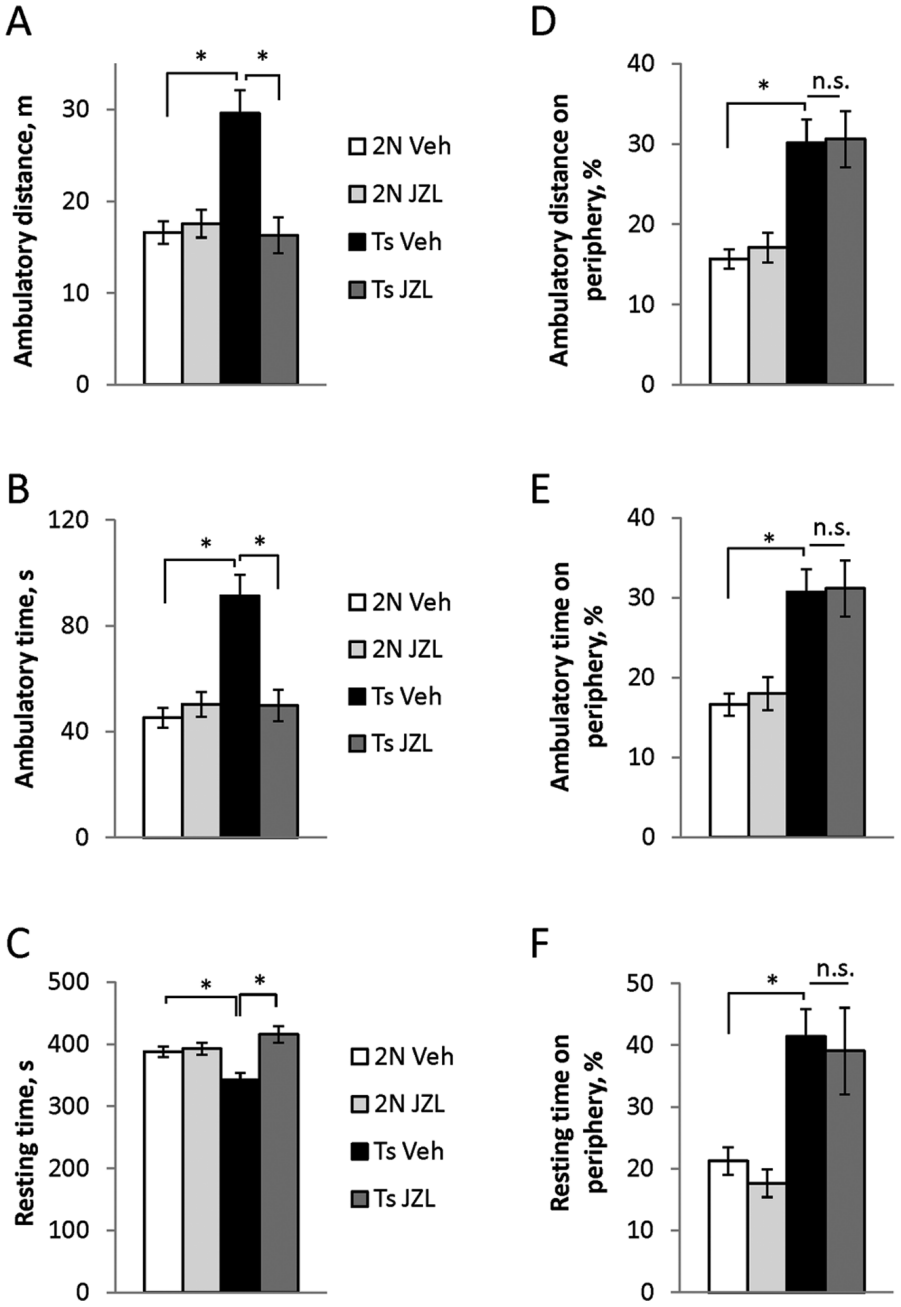
Effects of ZL184-treatment on locomotor activity (A-C) and thigmotactic behavior (D-F). In the vehicle-treated animals, locomotor activity was significantly increased in Ts65Dn vs. 2N mice. This can be seen from the increased ambulatory distance (A) and ambulatory time (B) and decreased resting time (C) of Ts Veh vs. 2N Veh group. Treatment with JZL184 (8 mg/kg) restored these locomotion parameters to control levels. In addition, Ts65Dn mice exhibited increased thigmotactic behavior which can be seen from an increased percentage of ambulatory distance (D), ambulatory time (E), and resting time (F) on the arena periphery. JZL184 treatment had no effect on these parameters.

Increased spontaneous locomotor activity is one of the most notable phenotypes of Ts65Dn mice [19], [38], [39], [40]. Similar to previous observations in younger animals, aged Ts65Dn mice showed considerably increased spontaneous locomotion. Both ambulatory distance and ambulatory time were almost two times greater, while the resting time was smaller in the vehicle-treated Ts65Dn vs. 2N mice (p<0.001) (Fig. 1 A-C). Remarkably, treatment with JZL184 fully normalized locomotor activity in Ts65Dn mice, while locomotor activity in 2N mice was not affected by the treatment (p = 0.2-0.8 for 2N Veh vs. 2N JZL groups) (Fig. 1 A-C). Thus, chronic treatment with JZL184 selectively altered the behavior of Ts65Dn mice, reducing abnormally elevated spontaneous locomotor activity to normal levels seen in 2N mice.

The exploratory habits of rodents are characterized by ‘thigmotactic behavior’, i.e. a desire to spend more time near vertical walls, which could be considered as an index of anxiety. Compared to 2N controls, vehicle-treated Ts65Dn mice travelled greater distances and spent a greater percentage of ambulatory and resting time on the arena periphery (Fig. 1D-F) thereby exhibiting increased thigmotactic behavior. At the drug dose tested (8 mg/kg), thigmotactic behavior was not affected by the treatment in both Ts65Dn and 2N mice (Fig. 1 D-F).

Since anxiety is affected by levels of AEA [41], we used an additional cohort of mice to examine the effects of a higher JZL184 dose (40 mg/kg), which increased levels of both 2-AG and AEA (Table 1). Similar to our earlier results, JZL184 treatment restored parameters of locomotor activity in Ts65Dn mice, but had no effect on the locomotion of 2N mice. However, in contrast to the lower dose, the higher JZL184 dose affected thigmotactic behavior of Ts65Dn mice. Thus, the percentage of the ambulatory distance, ambulatory time, and resting time spent on the arena periphery was reduced in the JZL184-treated Ts65Dn mice to the levels observed in 2N mice (p = 0.32-0.78 for Ts JZL vs. 2N Veh groups) (Fig. S1). Thigmotactic behavior of 2N control mice was not affected by the treatment.

#### Working memory

**Figure 2:**
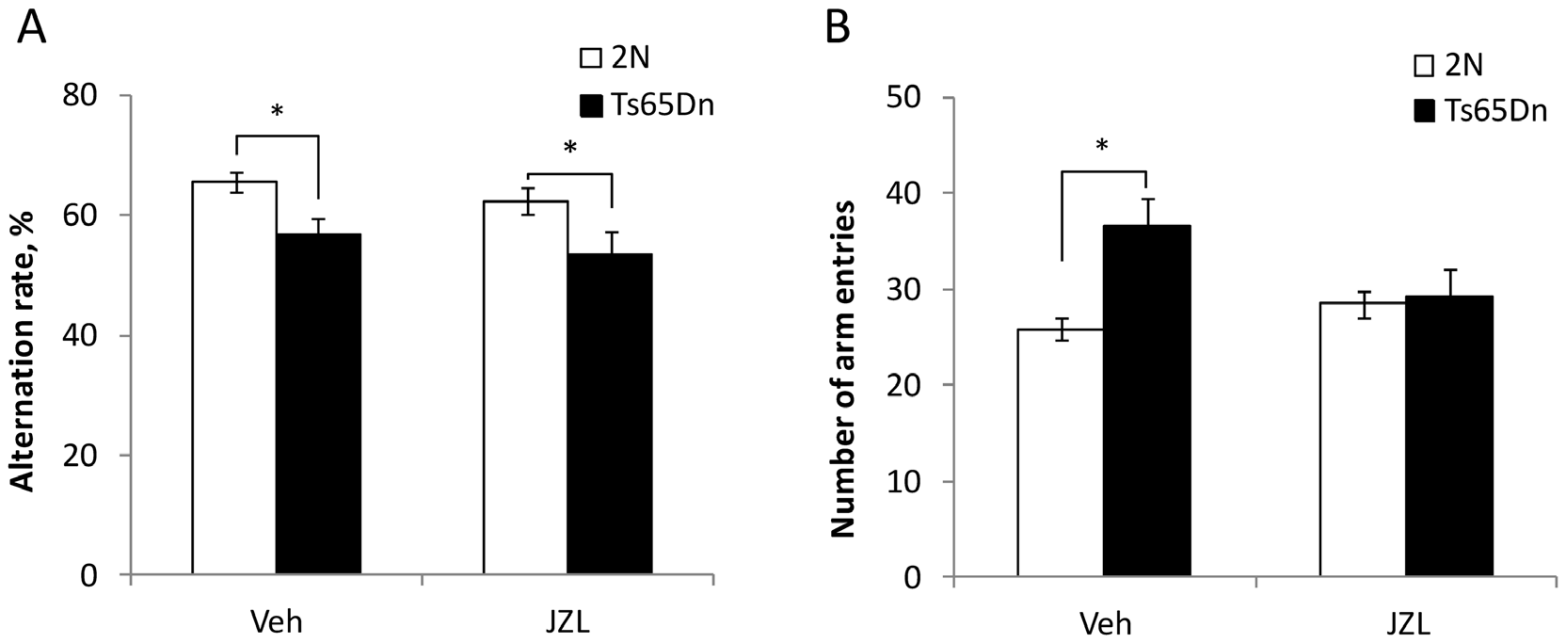
Working memory: performance in Y-maze. A. The baseline rate of spontaneous alternations in Y-maze was lower in the vehicle-treated Ts65Dn vs. 2N mice, reflecting an impairment of working memory. JZL184-treatment had no effect on the performance of both 2N and Ts65Dn mice suggesting no effect on working memory. B. The number of ‘arm entries’ during the Y-maze test was greater in vehicle-treated Ts65Dn vs. 2N mice reflecting increased locomotion of Ts65Dn mice. JZL184-treatment reduced this parameter to the levels seen in 2N animals.

Working memory was assessed by measuring the rate of spontaneous alternations in the Y-maze. The alternation rate was considerably lower in the vehicle-treated Ts65Dn vs. 2N mice (p<0.01), reflecting an impairment of working memory in aged Ts65Dn mice (Fig. 2A, *Veh*). JZL184 treatment did not affect the alternation rate in either 2N (p = 0.25 for JZL vs. Veh) or Ts65Dn (p = 0.77 for JZL vs. Veh) mice (Fig. 2A, *JZL*). Thus, suppression of MAGL activity did not improve working memory in aged Ts65Dn mice.

In addition to working memory, testing in the Y-maze allows for an independent estimation of spontaneous locomotor activity. The number of arm entries during the test was considerably greater in the vehicle-treated Ts65Dn vs. 2N mice (p<0.01), reflecting increased spontaneous locomotion (Fig. 2B, *Veh*). Treatment with JZL184 reduced the number of arm entries of Ts65Dn mice (p<0.03 for JZL vs. Veh), but had no effect on 2N controls (p = 0.17 for JZL vs. Veh) (Fig. 2B, *JZL*). Thus, JZL184-treatment did not affect working memory, but attenuated the elevated spontaneous locomotor activity of Ts65Dn mice, consistent with the effects seen in the activity chamber (Fig. 1).

#### Short-term memory

**Figure 3:**
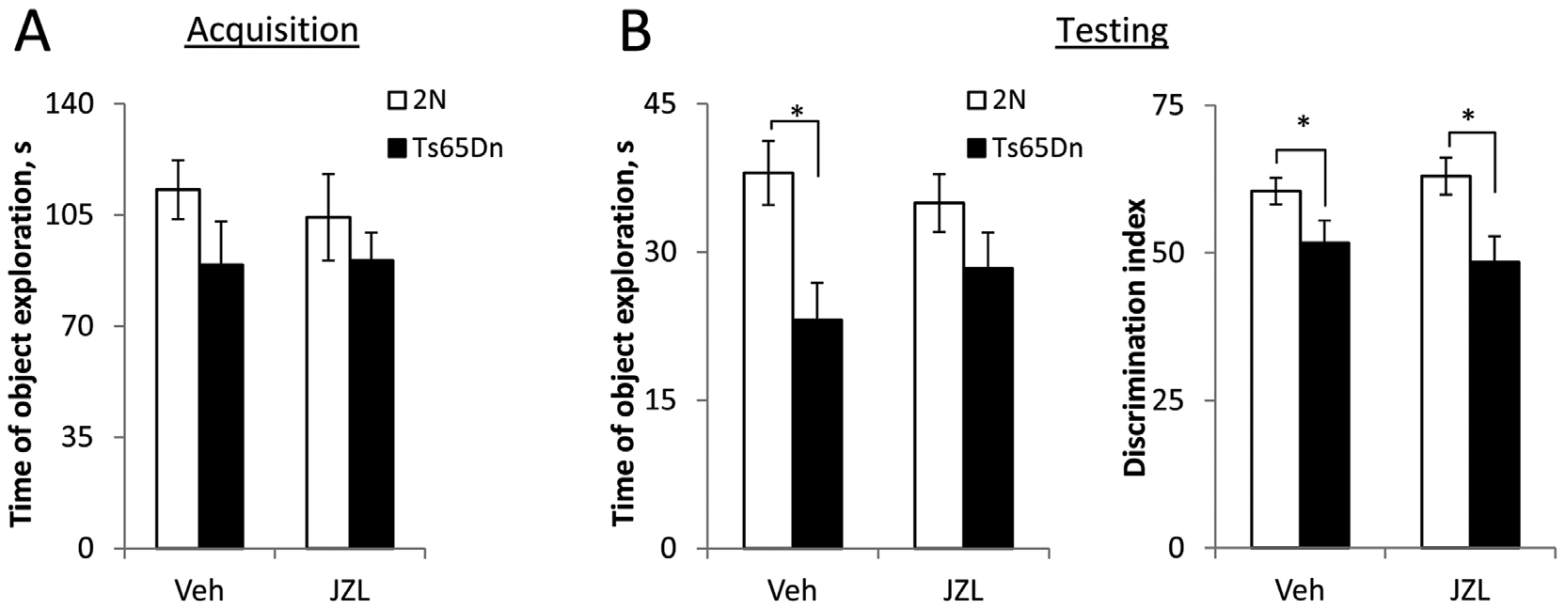
Short-term memory: Novel place recognition with the retention period of 10 min. A: Time spent investigating objects during acquisition. There was no difference between the groups. B: Testing phase. Left: Time spent investigating objects during testing. Vehicle-treated Ts65Dn mice spent less time investigating the objects than their littermate 2N controls. There was no such difference in the JZL-treated groups. Right: Discrimination index was smaller in both vehicle- and JZL-treated Ts65Dn vs. 2N groups. Thus, short-term memory was not affected by the JZL184-treatment.

The effect of JZL184-treatment on short-term memory was assessed in the novel place recognition test with a retention period of 10 min. In previous studies, this test showed a considerable impairment of short-term memory in younger Ts65Dn mice [19]. Here we observed that all groups of mice spent equal time exploring the objects during the acquisition phase (p = 0.3-0.8) (Fig. 3A). However, during the testing phase the time of object exploration was significantly smaller in vehicle-treated Ts65Dn vs. 2N mice (p<0.01) (Fig. 3B, left graph, *Veh*). The discrimination index was lower in Ts65Dn vs. 2N mice for both the vehicle-treated (p = 0.04) and the JZL184-treated (p = 0.008) mice (Fig. 3B, right graph). Comparisons between the JZL184- and vehicle-treated groups showed that the treatment had no effect in both Ts65Dn (p = 0.57) and 2N (p = 0.51) mice. Hence, similar to working memory, short-term memory was not affected by the JZL184-treatment.

#### Long-term memory

**Figure 4:**
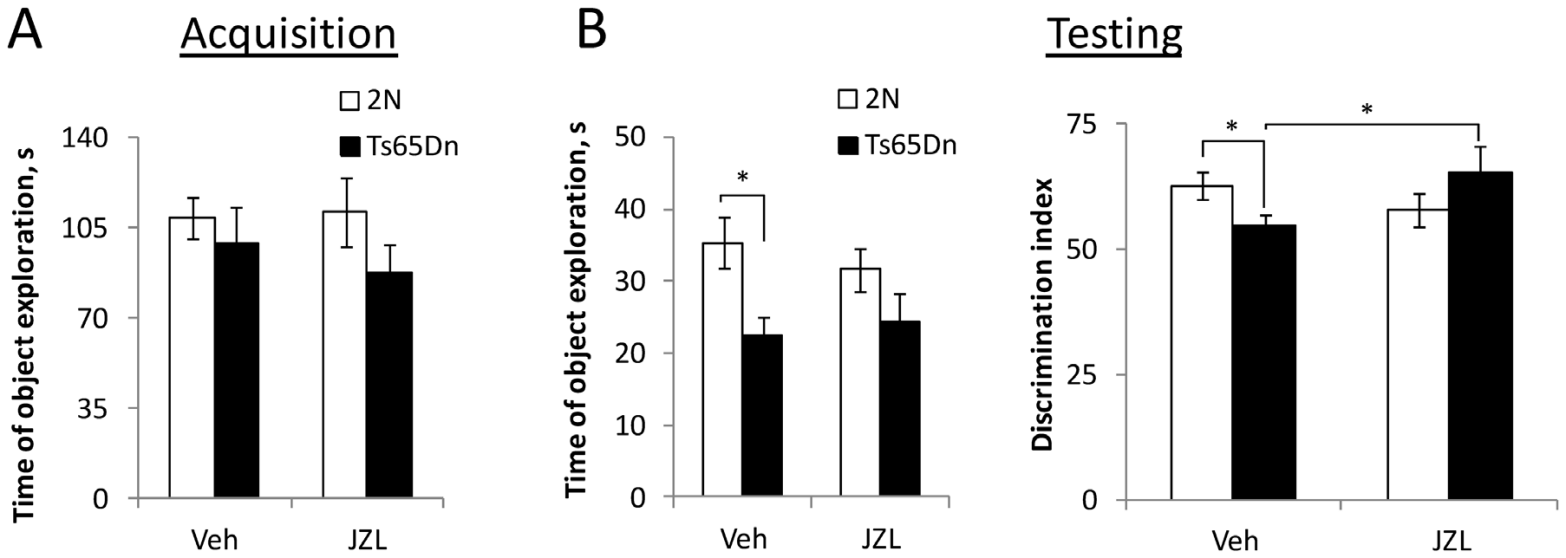
Long-term memory: Novel object recognition with the retention period of 24 hours. A: Time of object exploration during acquisition. There was no difference between the groups, and no effect of JZL184-treatment on the exploration time. B: Testing phase. Left: Time of object exploration during testing was smaller in vehicle-treated Ts65Dn vs. 2N mice. There was no such difference between the JZL184-treated Ts65Dn and 2N groups. Right: Discrimination index was smaller in the vehicle-treated Ts65Dn vs. 2N mice. JZL184-treatment significantly increased the discrimination index in Ts65Dn mice, but had no effect on the performance of their 2N littermates.

Deficiency of long-term memory is evident in people with DS, as well as in genetic models of DS. To assess long-term memory in aged Ts65Dn mice, we used the novel object recognition test with a retention period of 24 h. Previously, this test showed a reduction of long-term memory in young Ts65Dn mice [19], [42]. On average, all groups of mice spent equal time investigating the objects during the acquisition phase on Day1 (p = 0.3–0.75; Fig. 4A). However, similar to NPR, the time of object exploration during the testing phase on Day 2 was lower in Ts65Dn vs. 2N vehicle-treated mice (Fig. 4B, left graph, Veh). This difference was mostly due to a decrease in the time spent near the new objects. Consequently, the discrimination index was smaller in Ts65Dn vs. 2N vehicle-treated group (p = 0.048) (Fig. 4B, right graph), suggesting a deficiency of long-term recognition memory in aged Ts65Dn mice. Treatment with JZL184 improved performance of Ts65Dn mice (p = 0.037 for JZL vs. Veh). In fact, performance was not different in Ts65Dn JZL vs. 2N Veh mice (p = 0.59). Performance of 2N mice was not affected by the treatment (p = 0.45 for JZL vs. Veh). Thus, JZL184 treatment restored long-term memory in Ts65Dn mice but had no effect on 2N mice.

### Synaptic function and molecular characterization

#### Long-term potentiation

**Figure 5:**
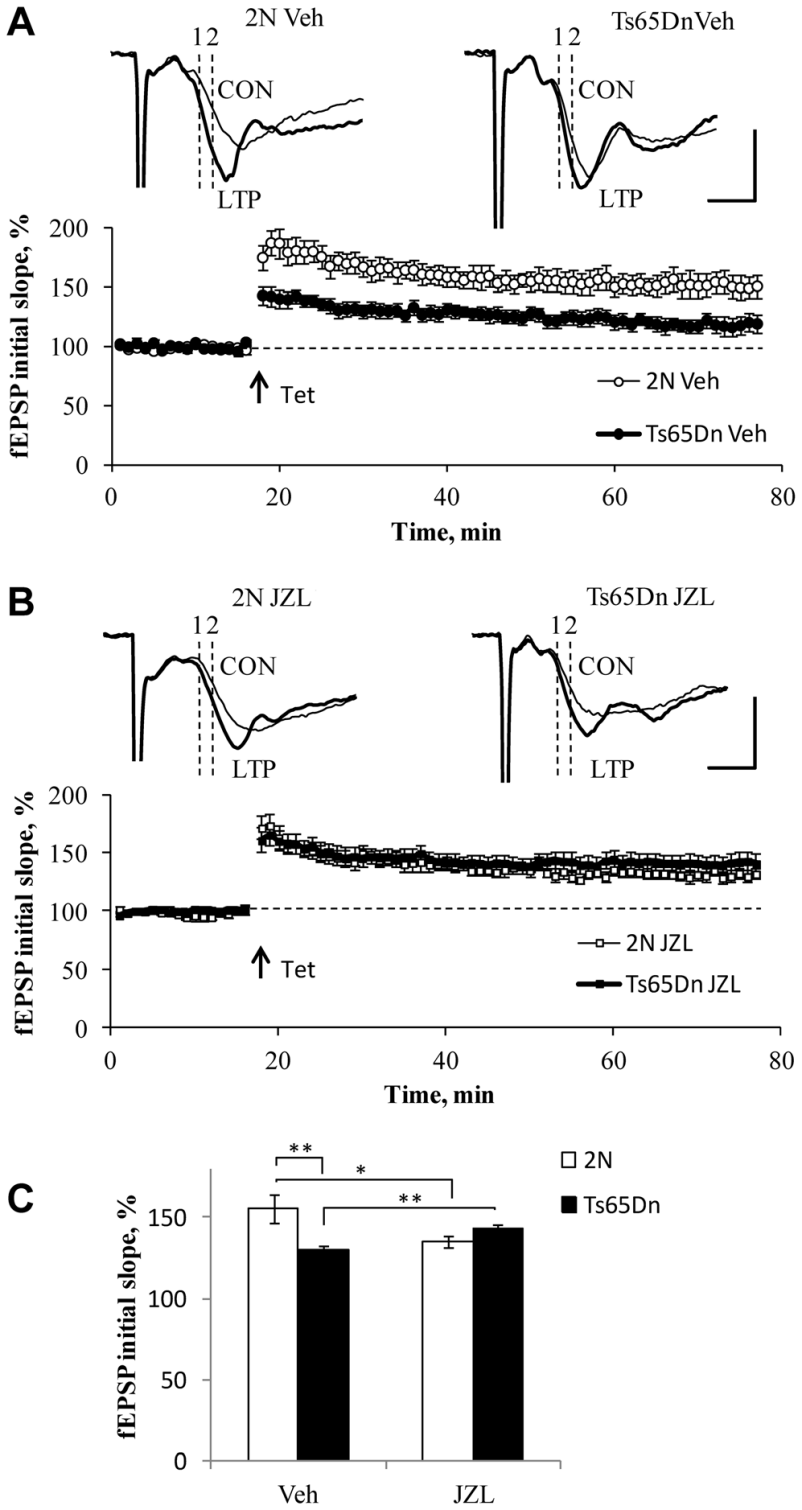
Long-term potentiation in the CA1 region of hippocampal slices of Ts65Dn and 2N mice. A: In the vehicle-treated animals, LTP was smaller in Ts65Dn vs. 2N slices. Scale bars: 1 mV; 2 ms. B: In the JZL184-treated mice, there was no difference between the Ts65Dn vs. 2N slices. C: Quantification of the data. JZL184-treatment increased LTP in Ts65Dn mice.

Changes in the behavior and memory of JZL184-treated Ts65Dn mice suggest that synaptic properties may also be affected. To assess the synaptic properties, a sub-group of mice used in the behavioral studies was examined in electrophysiological experiments using hippocampal slices. To assess long-term synaptic plasticity, LTP in the CA1 region was examined. LTP was significantly smaller in slices from vehicle-treated Ts65Dn vs. 2N mice (Fig. 5A, C). JZL184-treatment increased LTP in Ts65Dn mice (p = 0.005 for JZL vs. Veh) (Fig. 5B, C). Surprisingly, the treatment did not increase, but rather reduced LTP in 2N mice (p = 0.027 for JZL vs. Veh) (Fig. 5, A-C). As a result, LTP levels were not different in the JZL184-treated Ts65Dn and 2N mice (p = 0.18). Thus, following the JZL184 treatment, LTP was increased in Ts65Dn but reduced in 2N mice.

#### Levels of Aβ40 and Aβ42

**Figure 6:**
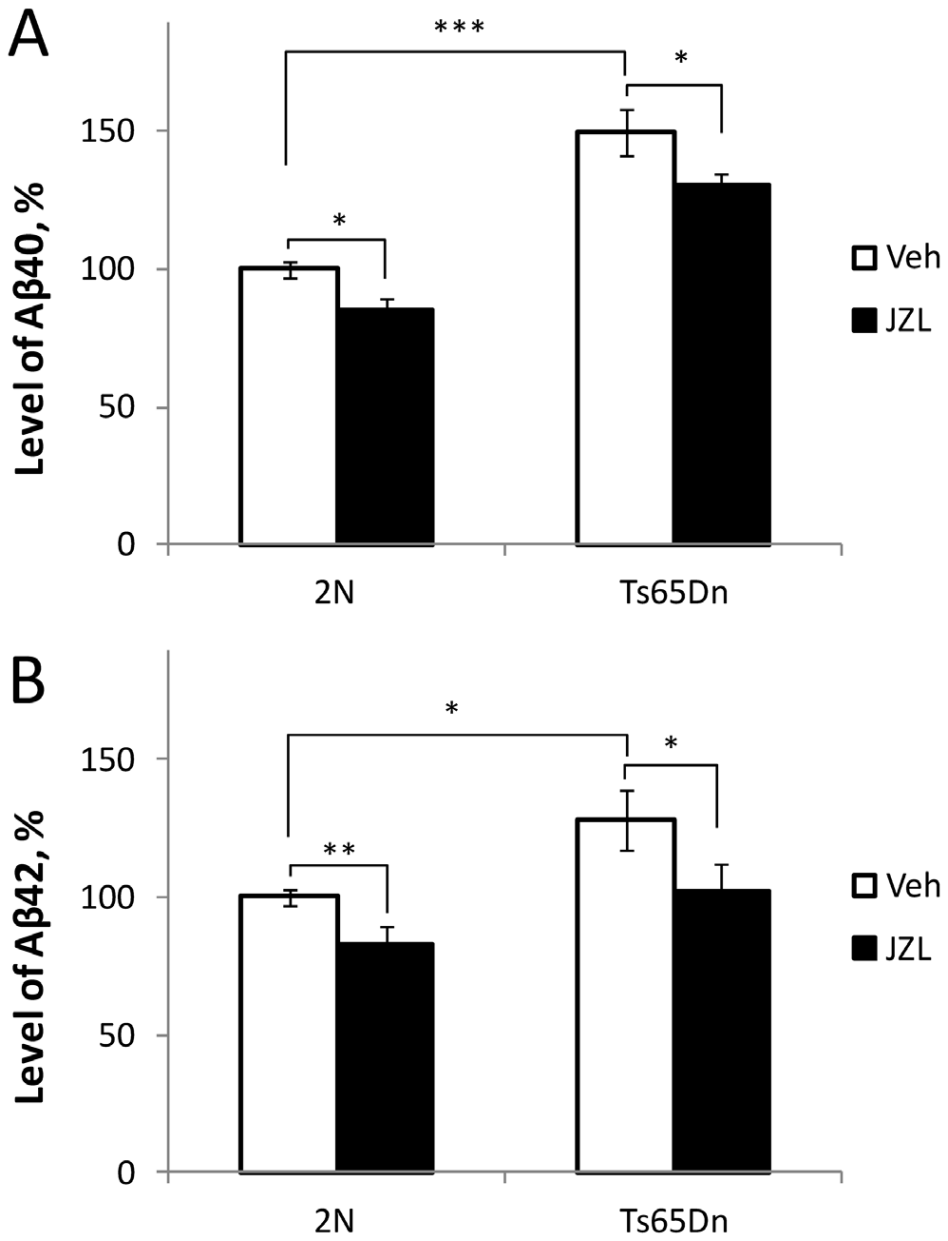
Levels of Aβ species in brain samples of mice treated with JZL184 or vehicle. A: Levels of Aβ40 were significantly increased in the vehicle-treated Ts65Dn vs. 2N mice. JZL184-treatment reduced the levels of Aβ40 in both Ts65Dn and 2N samples. B: Levels of Aβ42 were also greater in Ts65Dn vs 2N samples and were reduced by the JZL184-treatment.

Levels of Aβ40 and Aβ42 were measured in the brain samples of 2N and Ts65Dn mice using multi-array MSD technology. In the vehicle-treated animals, Ts65Dn mice had increased levels of both Aβ40 (2N: 10.1±0.8 pg/mL, n = 8; Ts65Dn: 15.2±1.4 pg/mL, n = 7; p = 0.005) and Aβ42 (2N: 0.58±0.02 pg/mL, n = 6; Ts65Dn: 0.74±0.07 pg/mL, n = 7; p = 0.031). Treatment with JZL184 significantly reduced levels of both Aβ40 and Aβ42 in Ts65Dn and 2N mice (Fig. 6). Expressed in percentage points of the corresponding vehicle-treated groups, the reduction was similar in 2N and Ts65Dn mice (Aβ40: 2N = 15.0±1.4%; Ts65Dn = 13.2±3.3%; p = 0.63; Aβ42: 2N = 17.1±6.5%; Ts65Dn = 20.3±6.1%; p = 0.66). Thus, chronic treatment with JZL184 reduced levels of Aβ40 and Aβ42 in both Ts65Dn and 2N mice.

#### Expression of proteins

**Figure 7:**
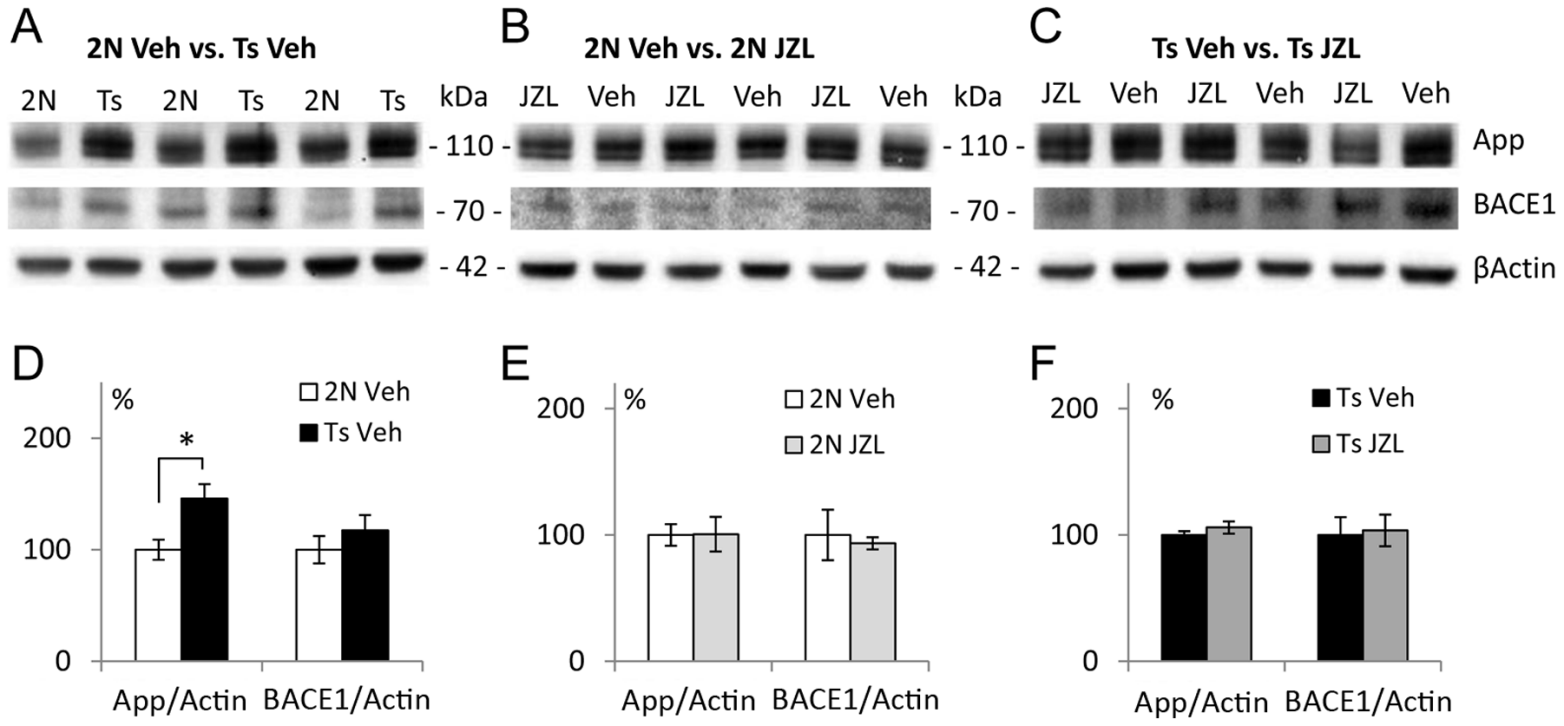
Levels of proteins. A –C: Examples of Western blots for between-group comparison of 2N Veh vs. Ts Veh, 2N Veh vs. 2N JZL, and Ts Veh vs. Ts JZL. D –F: Quantification of the data. Levels of App were increased, while the levels of BACE1 were not altered in the vehicle-treated Ts65Dn vs. 2N brain (A, D). Treatment with JZL184 had no effect on the expression levels of these proteins in either 2N (B, E) or Ts65Dn (C, F).

To assess the effects of JZL184-treatment on proteins relevant to the development of AD, we also assessed levels of App and BACE1 using Western blot (Fig. 7; Fig. S2). As expected, levels of App were increased in Ts65Dn vs. 2N mice approximately proportionally to the increase of the gene dose (50%, p = 0.013) (Fig. 7 A, C; Fig. S2). Although slightly higher, levels of BACE1 were not statistically different in the Ts65Dn vs. 2N samples (p = 0.34) (Fig. 7 A, C; Fig. S2). Treatment with JZL184 had no effect on the levels of either App (p = 0.26) or BACE1 (p = 0.17) in Ts65Dn mice (Fig. 7 B, C). This data suggests that the mechanisms of Aβ40 and Aβ42 decrease in Ts65Dn mice by JZL184 treatment do not rely on reduced expression of full length App or BACE1 proteins.

## Discussion

The aim of this study was to examine the effects of chronic inhibition of MAGL, the key hydrolytic enzyme of endocannabinoid 2-AG, on the behavioral and neural properties of Ts65Dn mice, an animal model of DS_._ We observed that chronic treatment with the selective MAGL inhibitor JZL184 reduced spontaneous locomotor activity to normal, improved long-term memory, and increased hippocampal long-term potentiation in Ts65Dn mice. These measures were not affected in the normosomic control mice suggesting that MAGL inhibition is especially effective in the trisomic animals. The JZL184-treatment also reduced levels of Aβ40 and Aβ42 equally in both Ts65Dn and 2N mice, but it had no effect on full length App and BACE1. These data show that chronic suppression of MAGL activity may improve behavior and brain functions of Ts65Dn mice and, therefore, pharmacological inhibition of MAGL may be a new approach for the pharmacotherapy of cognitive impairment in DS.

The endocannabinoid system plays an important role in many brain functions including locomotion, emotions, and cognition [43], [44]. 2-AG and AEA are the principal endogenous ligands of cannabinoid receptors [1], [45]. Unlike traditional neurotransmitters, endocannabinoids are lipophilic and cannot be stored in membrane-enclosed secretory vesicles. Therefore, their levels are controlled by a balance of on-demand biosynthesis and enzymatic degradation. In the central nervous system, degradation of 2-AG is primarily caused by MAGL [2], genetic ablation or pharmacological blockade of which results in notable alterations in synaptic plasticity and behavior [4], [7], [8]. Recently, it was shown that chronic inhibition of MAGL with JZL184 in 5XFAD mice, a model of rapid brain amyloidosis co-expressing 5 mutations of familial AD [46], increased hippocampal LTP and improved learning [7]. Unlike the AD models, Ts65Dn mice have no mutated genes but contain one extra copy of several hundred of genes, including the *App* gene. As a result, Ts65Dn mice show a number of AD features later in life. Thus, at the age of 6 months Ts65Dn mice show a degeneration of basal forebrain cholinergic neurons (BFCNs) [30], [31], a prominent pathological feature contributing to cognitive dysfunction in AD. Protein expression analysis in hippocampal CA1 pyramidal neurons of aged Ts65Dn mice revealed a decrease in the levels of several neurotrophins and their cognate receptors, subunits of glutamatergic AMPA and NMDA receptors, and other changes [32] similar to those observed in AD [47], [48], [49], [50]. Thus, aged Ts65Dn mice exhibit many features found in AD and, therefore, can be regarded as a model for the development of AD in DS.

Baseline spontaneous locomotor activity was considerably increased in aged Ts65Dn vs. 2N control mice. Similar changes in locomotion were previously described in younger Ts65Dn mice [19], [38], , as well as in mouse AD models [51], [52], [53], [54]. In people with DS, hyperkinetic and hyperactivity disorders are common. For example, children with DS have considerably increased rates of hyperkinetic conduct disorder [55] and high prevalence for attention deficit hyperactivity disorder (ADHD) [56], [57]. Disinhibition and aberrant motor behaviors were also noted in AD [58], [59]. Thus, the increased spontaneous locomotor activity of Ts65Dn mice may be considered a model for the aberrant motor behaviors in DS and AD.

We observed that baseline levels of 2-AG were elevated in Ts65Dn vs. 2N mice. One possible explanation for this change is a compensatory reaction of neural tissue aimed at restoring brain functions during development. If so, further elevation of 2-AG levels with pharmacological agents could facilitate the restoration. The improvement of several types of behavior and synaptic plasticity in Ts65Dn mice observed in this study agrees with such a possibility.

JZL184-treatment reduced locomotion of Ts65Dn mice to the levels observed in their normosomic controls. Both the high and the low drug doses were equally effective suggesting that the primary cause of these effects was elevation of 2-AG, and that elevation of AEA, which was observed with the higher drug dose, had no additional effects. These results agree with the earlier findings that selective inhibitors of FAAH, which considerably increased brain levels of AEA, had no effects on locomotor activity in mice [60]. Interestingly, locomotion of normosomic mice was not affected by the JZL184-treatment at either dose. Previously, both a reduction [4] and an elevation [61] of locomotor activity have been reported after treatment with JZL184. Thus, the profound attenuation of the hyper locomotor activity observed in Ts65Dn mice, along with a lack of efficiency in aged normosomic mice, suggests that MAGL may mediate pathologies that are specific to DS.

Thigmotactic behavior, characterized by clinging to vertical walls, is generally considered as an index of anxiety [62]. Previously, we found no changes in this behavior in 3 month old Ts65Dn mice [19]. Here, however, we observed significantly increased thigmotaxis in the 12 month old Ts65Dn mice. This increase in anxiety-associated behavior may signify the presence of AD-type changes in older Ts65Dn mice. Indeed, increased thigmotactic behavior and increased anxiety were observed in a number of mouse AD models [63], [64], as well as in AD patients [65], [66]. Thigmotactic behavior of Ts65Dn mice was reduced to normal levels by the higher JZL184 dose, but it was unaffected by the lower dose. These findings suggest that elevation of AEA is responsible for the anxiolytic effects of JZL184 in Ts65Dn mice, while elevation of 2-AG alone has no effect, in agreement with previous observations. For example, Ts65Dn mice tested in the ‘marble burying’ assay, a test for compulsive behavior and anxiety, showed no effect with a low dose of JZL184 (4–8 mg/kg) but a decrease in burying activity with a higher dose (40 mg/kg), while genetic deletion or pharmacological inhibition of FAAH, which selectively increased levels of AEA, reduced anxiety-like behaviors in the elevated plus maze [67], [68], [69], zero maze [70], and light/dark box [41]. Thus, the fact that a reduction of anxiety-like behaviors in Ts65Dn mice could only be achieved with JZL184 doses that increased brain levels of AEA is consistent with a mechanism requiring FAAH inhibition.

Down syndrome, and possibly the subsequent progression of AD, negatively affects several types of memory. Working and short-term memory is impaired in DS [71] and AD [72], [73]. Impairment of hippocampus-dependent long-term memory is regarded as the major hallmark of AD, but this memory is also impaired in DS [22]. Accordingly, genetic models of DS and AD showed severe abnormalities in learning and memory [12], [38], [42], [74]. In agreement with those findings, here we also observed that working and long-term types of memory were impaired in aged Ts65Dn mice.

A number of mechanisms may contribute to the learning and memory abnormalities found in Ts65Dn mice. Previously, we and others showed that inhibitory neurotransmission is enhanced in Ts65Dn mice, and that this change restricts the activation of NMDA receptors leading to reduced LTP and deficient memory [75], [76], [77], [78], [79]. The imbalance between GABAergic and glutamatergic neurotransmission was also observed in mouse AD models [80], [81]. Consequently, a number of treatments restoring the inhibitory/excitatory balance improved both LTP and memory in Ts65Dn mice [19], [42], [82], [83], [84]. Here we observed that LTP and memory of aged Ts65Dn mice were improved by a drug which increased brain levels of 2-AG. Activation by 2-AG of presynaptic CB1 receptors affects the release of both GABA and glutamate, thus regulating the excitatory-inhibitory balance [85], [86]. Thus, it is possible that the improvements in synaptic plasticity and memory by JZL184-treatments rely, in part, on the regulation of the balance between excitatory and inhibitory neurotransmission. Also, it was recently observed that overexpression of *Dyrk1a*, one of the genes triplicated in Ts65Dn mice and DS, resulted in the ablation of endocannabinoid-mediated long-term depression in the prefrontal cortex, while JZL184 treatment restored this type of synaptic plasticity [87]. Effects of the JZL184 treatments on the excitatory/inhibitory balance in mouse models of DS and AD should be examined in future studies.

One additional notable outcome of the JZL-treatments was a reduction in brain levels of Aβ40 and Aβ42. Interestingly, such changes were equal in Ts65Dn and 2N mice, suggesting no effect of the baseline Aβ levels, which were greater in Ts65Dn vs. 2N mice, on the drug efficiency. Recently, it was shown that both genetic [9] and pharmacological [7] suppression of MAGL reduced the levels of Aβ in mouse AD models. Since increased Aβ levels negatively affect synaptic plasticity and memory, this change may have also contributed to the memory restoration observed in Ts65Dn mice. Thus, a number of mechanisms may have contributed to the restoration of synaptic plasticity and learning in the JZL184-treated Ts65Dn mice.

## Conclusions

We observed here that chronic inhibition of MAGL with the selective inhibitor JZL184 restored locomotor activity and improved long-term memory and synaptic plasticity in Ts65Dn mice, a model of DS_._ The JZL184-treatment had no effect on the behavior and synaptic plasticity of normosomic control mice suggesting that suppression of MAGL activity is specifically efficacious in DS. This treatment also reduced levels of Aβ40 and Aβ42 but had no effect on the levels of full length App and BACE1 in both the Ts65Dn and 2N mice. Together, these results suggest that pharmacological targeting of MAGL may represent a novel and specific therapeutic approach for improving the cognitive impairments in DS.

## Supporting Information

Figure S1 **Effect of a high JZL184 dose on locomotor activity and thigmotactic behavior.** Locomotor activity (A-C) and thigmotactic behavior (D-F) were measured in Ts65Dn and 2N mice after JZL184 treatment at a dose of 40 mg/kg. Both the locomotor activity and the thigmotactic behavior were significantly increased in the vehicle-treated Ts65Dn vs. 2N mice, as can be seen from the data for the ambulatory distance (A, D), the ambulatory time (B, E), and the resting time (C, F). JZL184 treatment restored these parameters in Ts65Dn mice to the levels observed in 2N mice. There was no effect of the JZL184-treatment on locomotion or thigmotactic behavior in 2N mice.
(TIF)

Figure S2 **Original Western blots.** A. Blot # 1 with alternatively placed samples of the vehicle-treated 2N and Ts65Dn mice. The blot was exposed to the rabbit polyclonal anti-App antibody (the first primary antibody). The part of the figure marked by the dotted rectangle is shown in Figure 7A as ‘App’. In addition, this and other blots were exposed to anti-BACE1 antibody (M-83, Santa Cruz, 1∶1000), which produced a band at ∼51 kDa, i.e. significantly lower than the expected for BACE1 value of ∼70 kDa. Since the correct identify of this band could not be confirmed with a blocking peptide, the results for this band were not include in the paper. Of note, evaluation of results for this band showed no effect of JZL184 treatment in both 2N and Ts65Dn samples, which is similar to the results observed for the 70 kDa band. B. Blot # 1 after exposure to the rabbit anti- βActin antibody (the second primary antibody). Lines for both App and βActin are visible. The part of the figure marked by the dotted rectangle is shown in Figure 7A as ‘βActin’. C. Blot # 1 after exposure to the rabbit anti- BACE1 antibody (the third primary antibody). Lines for BACE1, App, and App are visible. The part of the figure marked by the dotted rectangle is shown in Figure 7A as ‘BACE1’. D. Blot # 2 with alternatively placed samples of the vehicle- and JZL-treated 2N mice. The blot was exposed to the rabbit polyclonal anti-App antibody (the first primary antibody). The part of the figure marked by the dotted rectangle is shown in Figure 7B as ‘App’. E. Blot # 2 after exposure to the rabbit anti-βActin antibody (the second primary antibody). Lines for both App and βActin are visible. The part of the figure marked by the dotted rectangle is shown in Figure 7B as ‘βActin’. F. Blot # 2 after exposure to the rabbit anti-BACE1 antibody (the third primary antibody). Lines for BACE1, App, and App are visible. The part of the figure marked by the dotted rectangle is shown in Figure 7B as ‘BACE1’. G. Blot # 3 with alternatively placed samples of the vehicle- and JZL-treated Ts65Dn mice. The blot was exposed to the rabbit polyclonal anti-App antibody (the first primary antibody). The part of the figure marked by the dotted rectangle is shown in Figure 7C as ‘App’. H. Blot # 3 after exposure to the rabbit anti- βActin antibody (the second primary antibody). Lines for both App and βActin are visible. The part of the figure marked by the dotted rectangle is shown in Figure 7C as ‘βActin’. I. Blot # 3 after exposure to the rabbit anti- BACE1 antibody (the third primary antibody). Lines for BACE1, App, and App are visible. The part of the figure marked by the dotted rectangle is shown in Figure 7C as ‘BACE1’.
(PDF)

Table S1 **Effect of chronic JZL184 treatment on the brain levels of lipids in Ts65Dn vs. 2N mice.** Targeted metabolomic measurements were performed via multiple-reaction monitoring (MRM) mass spectrometry on lipid extracts from the brains of Ts65Dn and 2N mice treated with vehicle or JZL184. Metabolite levels are shown relative to the brains from 2N vehicle-treated mice. Two-way ANOVA F ratios and p values, as well as p-values for Student's two-tails T-test are shown and values of statistical significance (p<0.05) are shown in bold.
(DOCX)
