# Supplementary figures and images for: Monoacylglycerol Lipase Inhibitor JZL184 Improves Behavior and Neural Properties in Ts65Dn Mice, a Model of Down Syndrome
Source: PLoS One. 2014 Dec 4;9(12):e114521. doi: 10.1371/journal.pone.0114521 (PMC4256450; doi:10.1371/journal.pone.0114521)

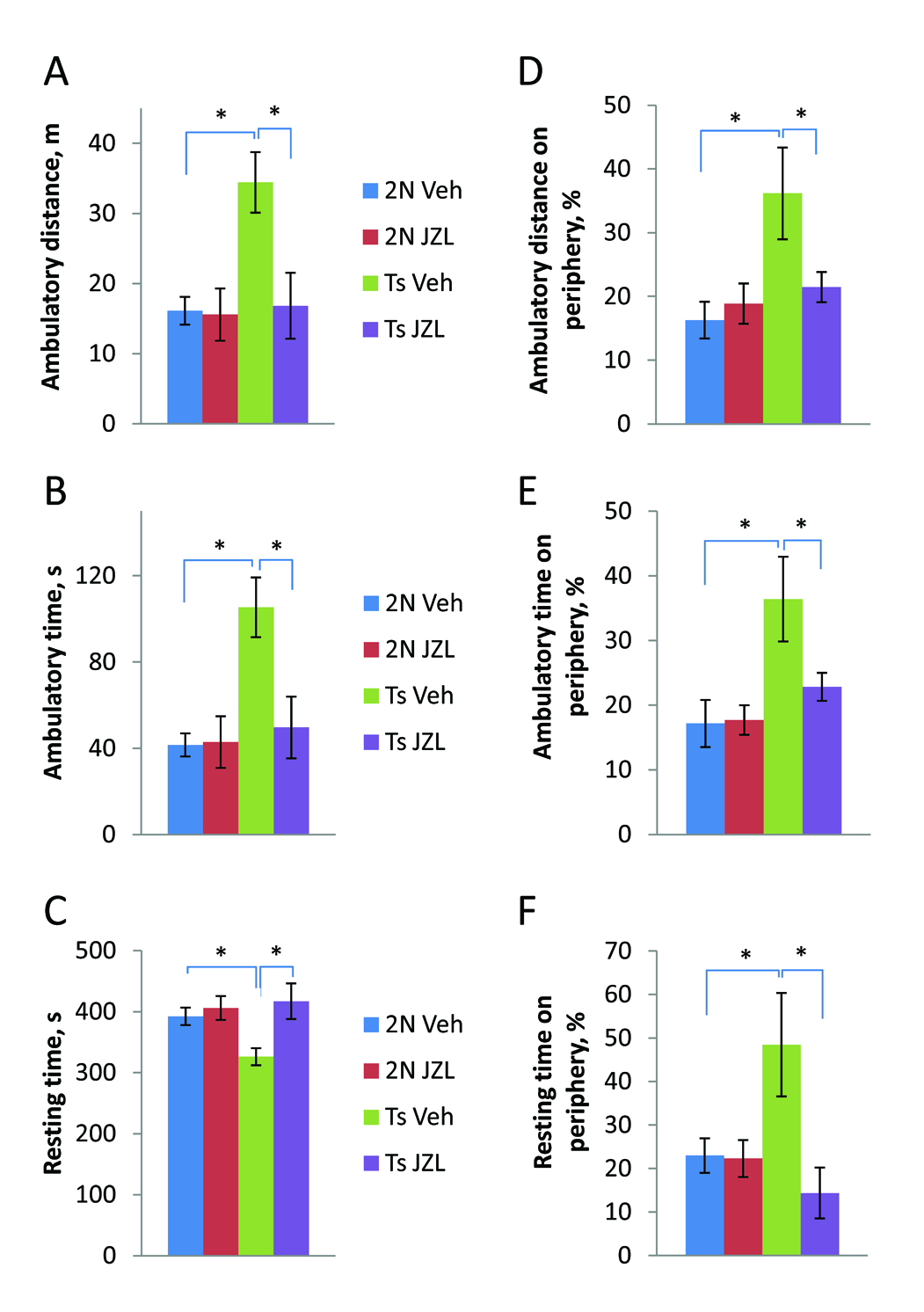

Supplement: Figure S1 — Effect of a high JZL184 dose on locomotor activity and thigmotactic behavior. Locomotor activity (A-C) and thigmotactic behavior (D-F) were measured in Ts65Dn and 2N mice after JZL184 treatment at a dose of 40 mg/kg. Both the locomotor activity and the thigmotactic behavior were significantly increased in the vehicle-treated Ts65Dn vs. 2N mice, as can be seen from the data for the ambulatory distance (A, D), the ambulatory time (B, E), and the resting time (C, F). JZL184 treatment restored these parameters in Ts65Dn mice to the levels observed in 2N mice. There was no effect of the JZL184-treatment on locomotion or thigmotactic behavior in 2N mice. (TIF) [file pone.0114521.s001.tif]
