# Supplementary material for: Monoacylglycerol Lipase Inhibitor JZL184 Improves Behavior and Neural Properties in Ts65Dn Mice, a Model of Down Syndrome
Source: PLoS One. 2014 Dec 4;9(12):e114521. doi: 10.1371/journal.pone.0114521 (PMC4256450; doi:10.1371/journal.pone.0114521)

Figure S2A. Blot 1 (Ts Veh vs. 2N Veh) after App antibody

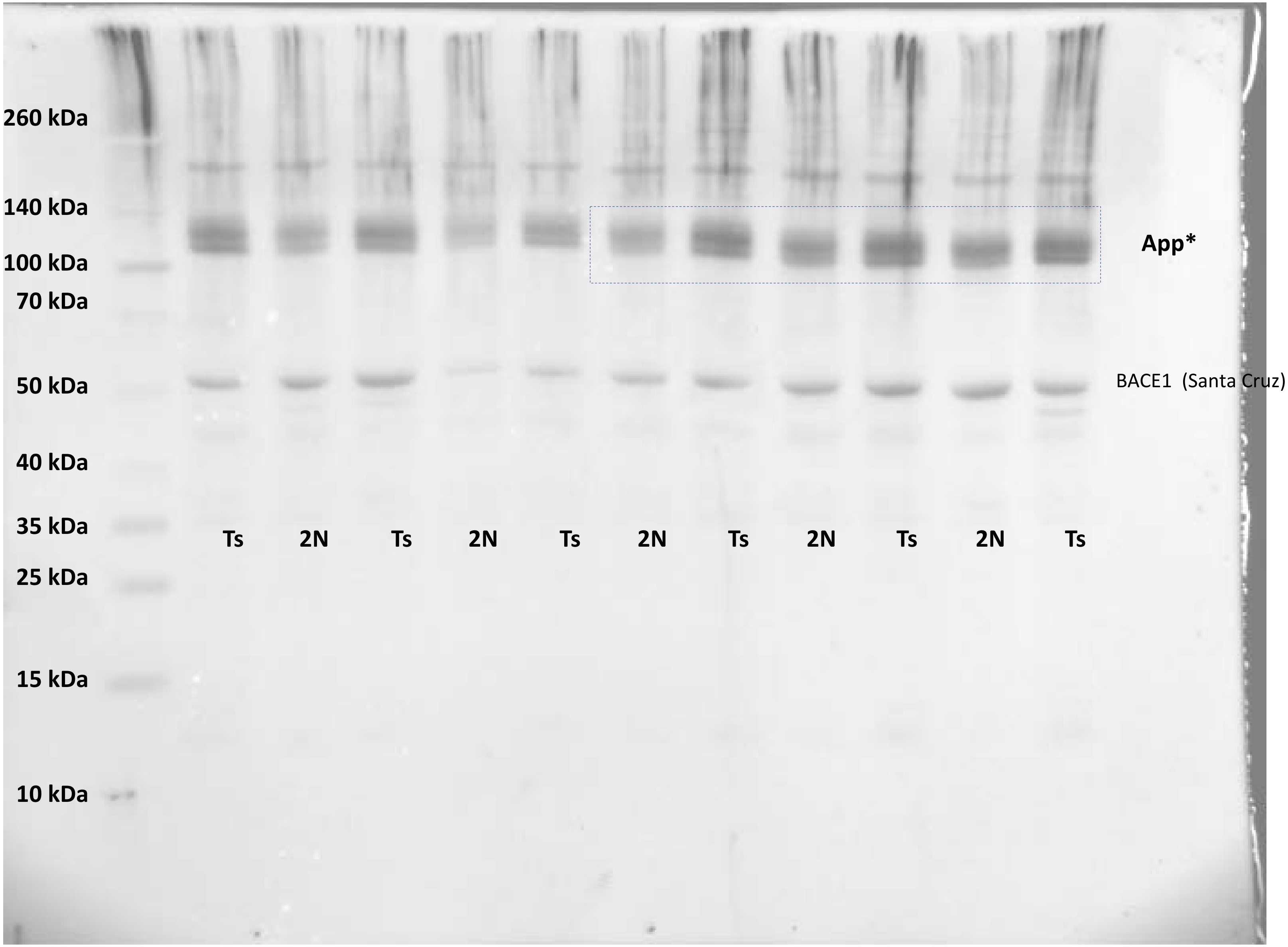

Figure S2B. Blot 1 (Ts Veh vs. 2N Veh) after App + βActin antibodies

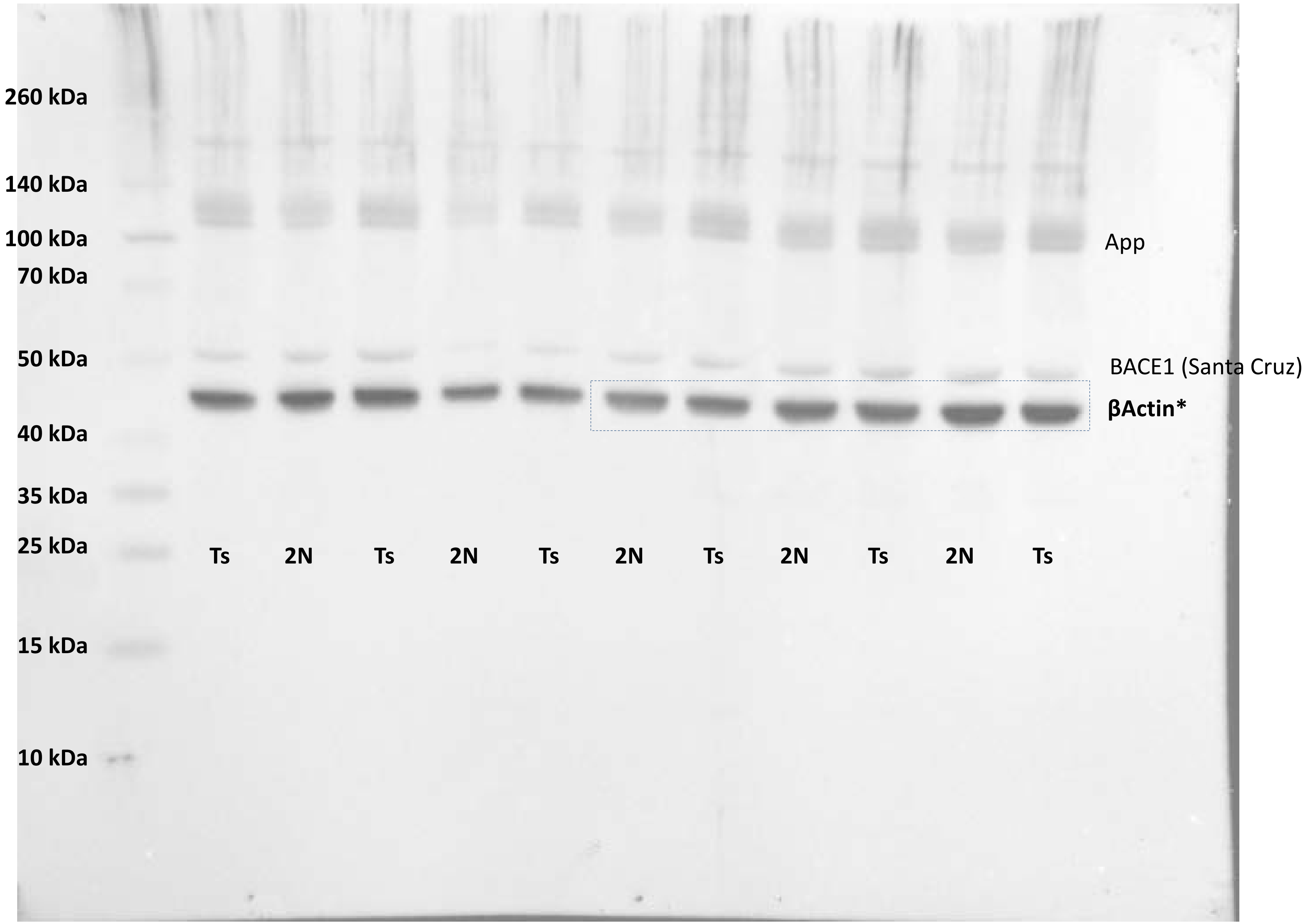

Figure S2C. Blot 1 (Ts Veh vs. 2N Veh) after App + βActin + BACE1 antibodies

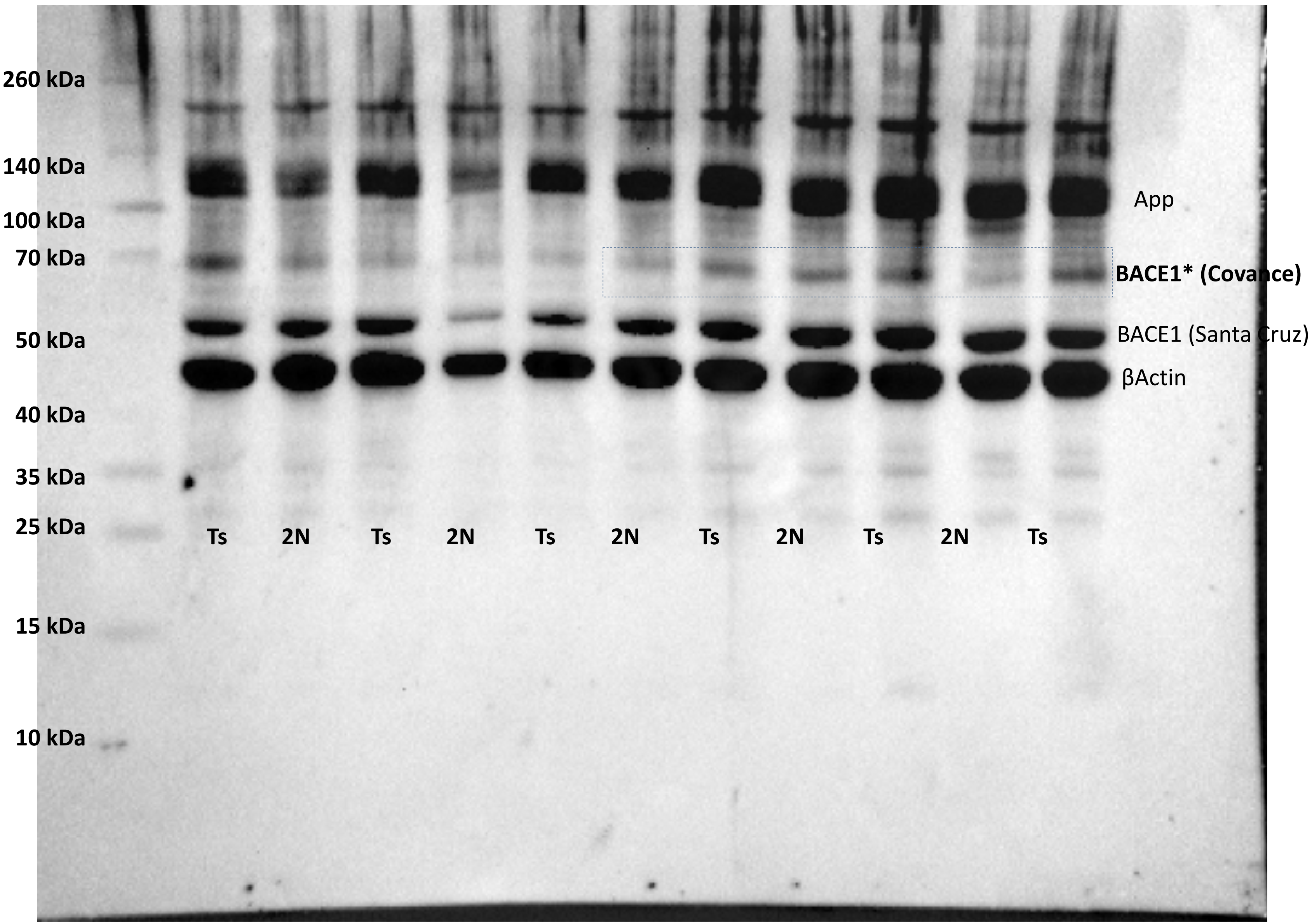

Figure S2D. Blot 2 (2N Veh vs. 2N JZL) after App antibody

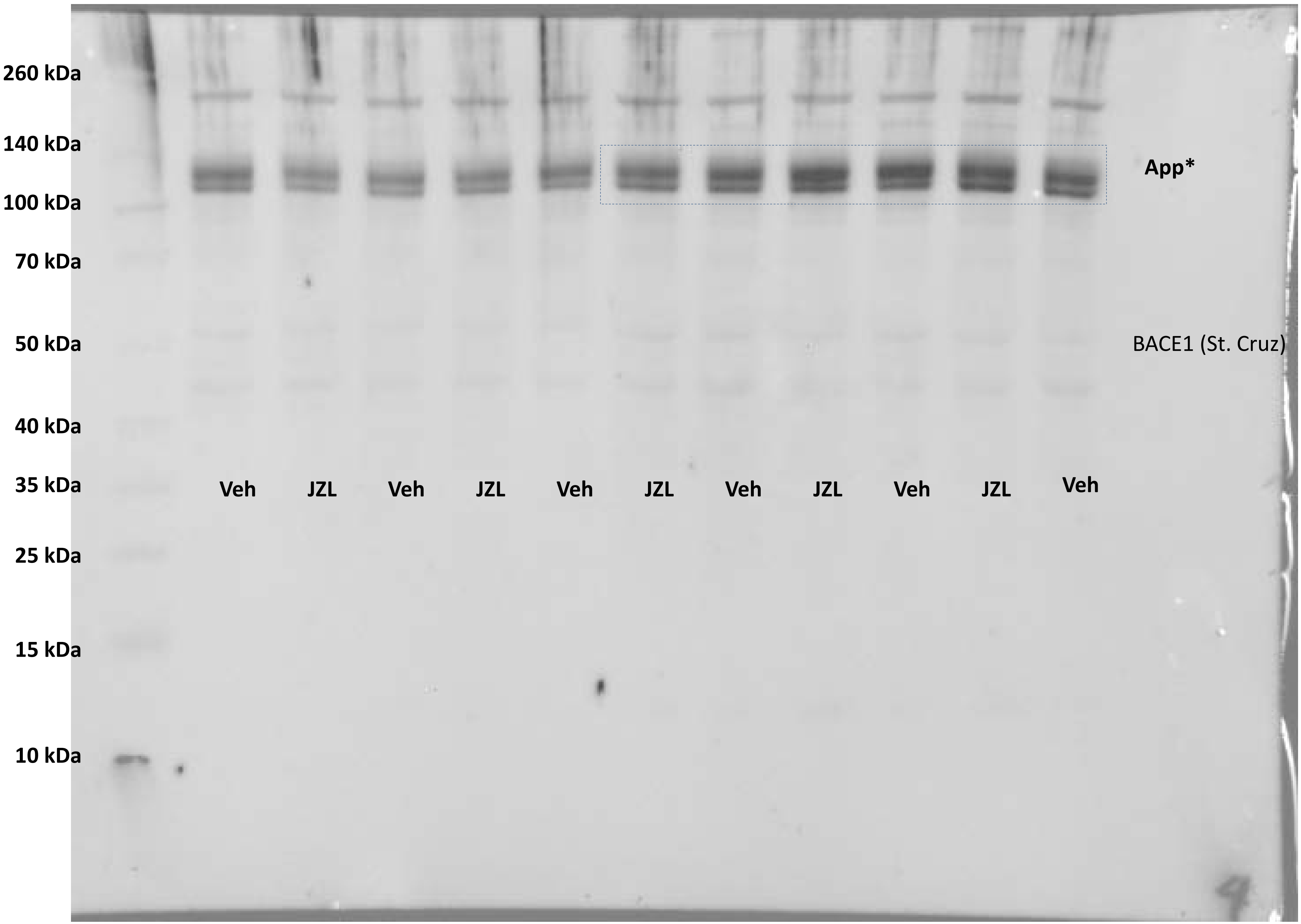

Figure S2E. Blot 2 (2N Veh vs. 2N JZL) after App+ βActin antibodies

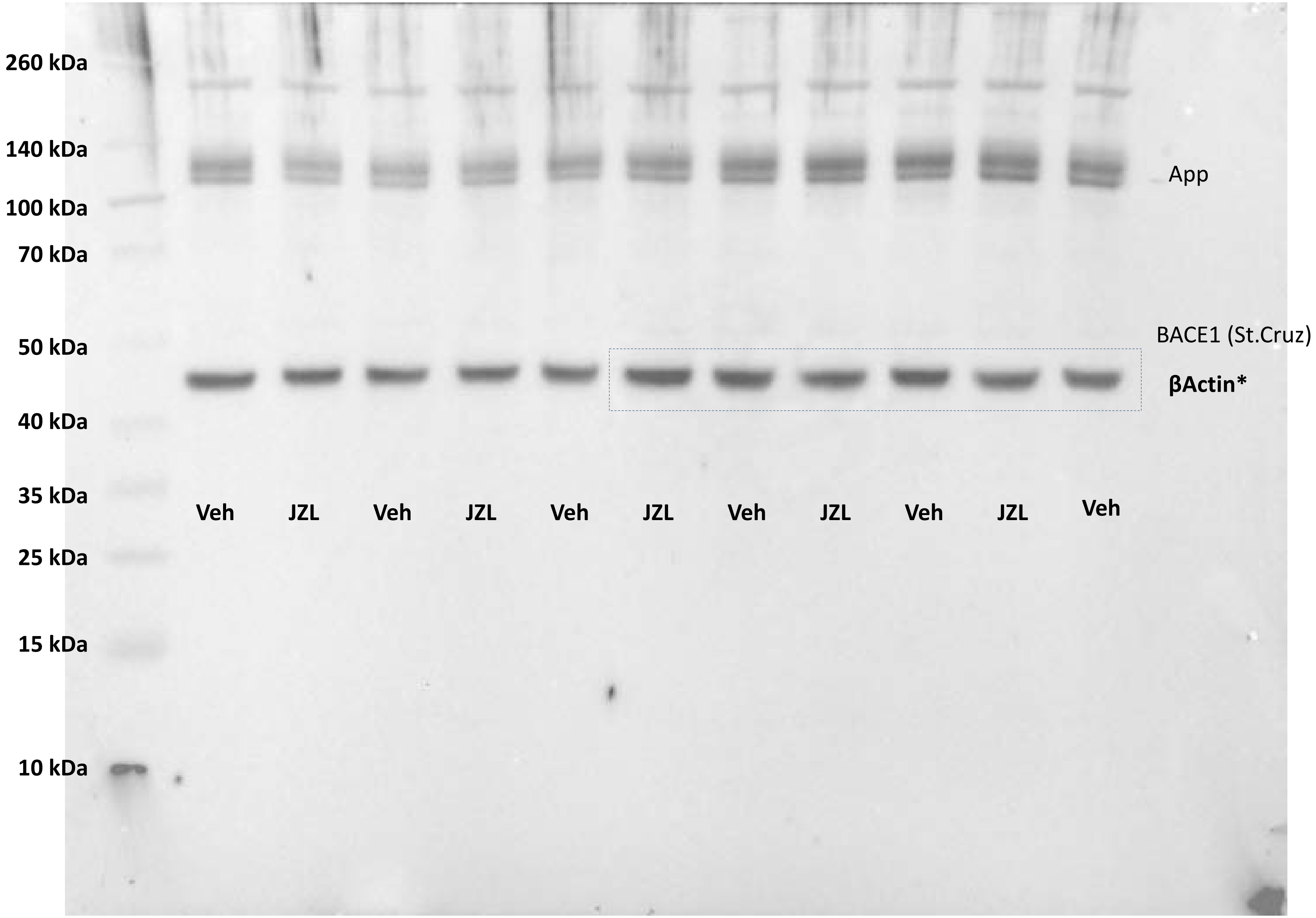

Figure S2F. Blot 2 (2N Veh vs. 2N JZL) after App+ βActin antibodies

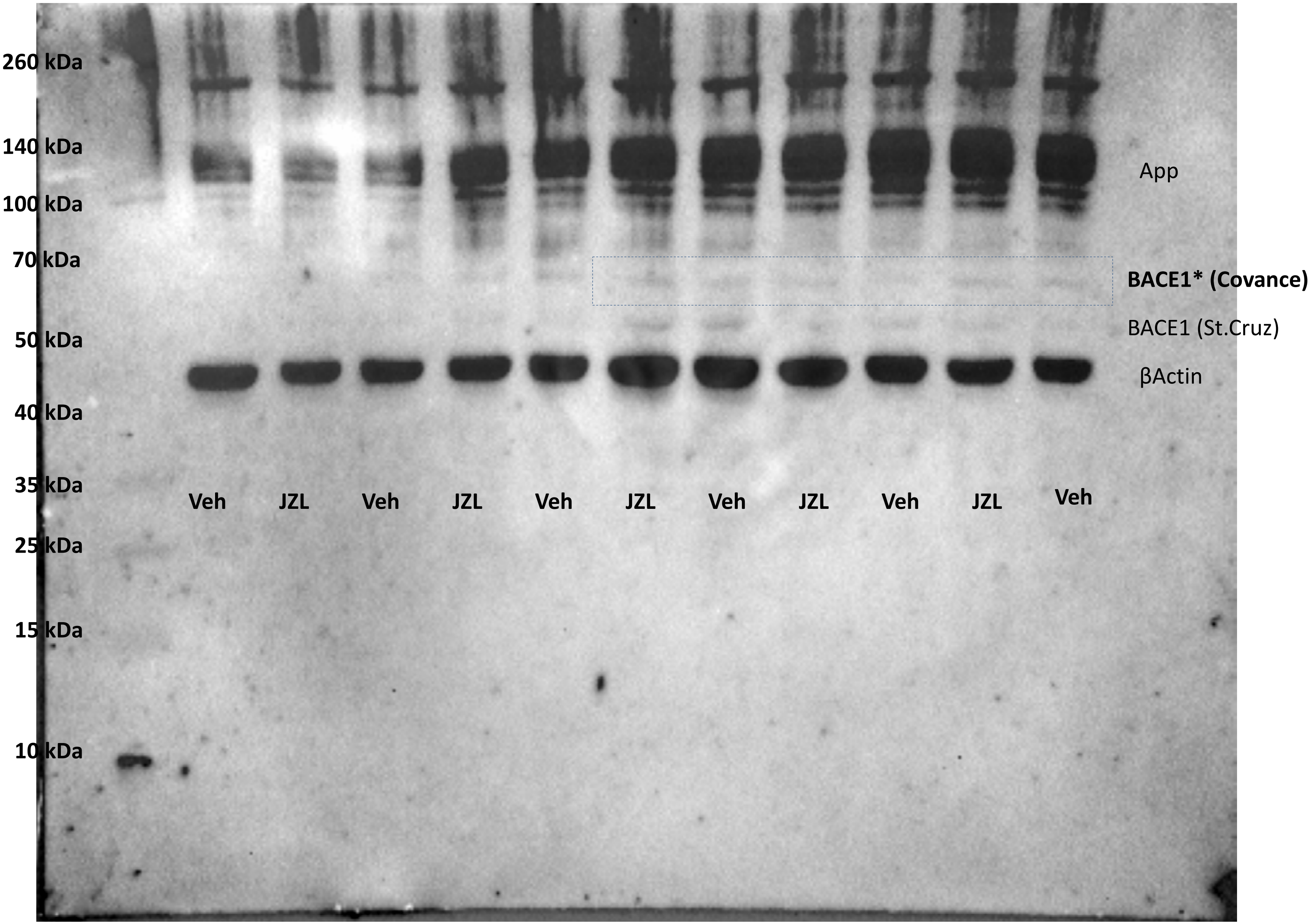

Figure S2G. Blot 3 (Ts Veh vs. Ts JZL) after App antibody

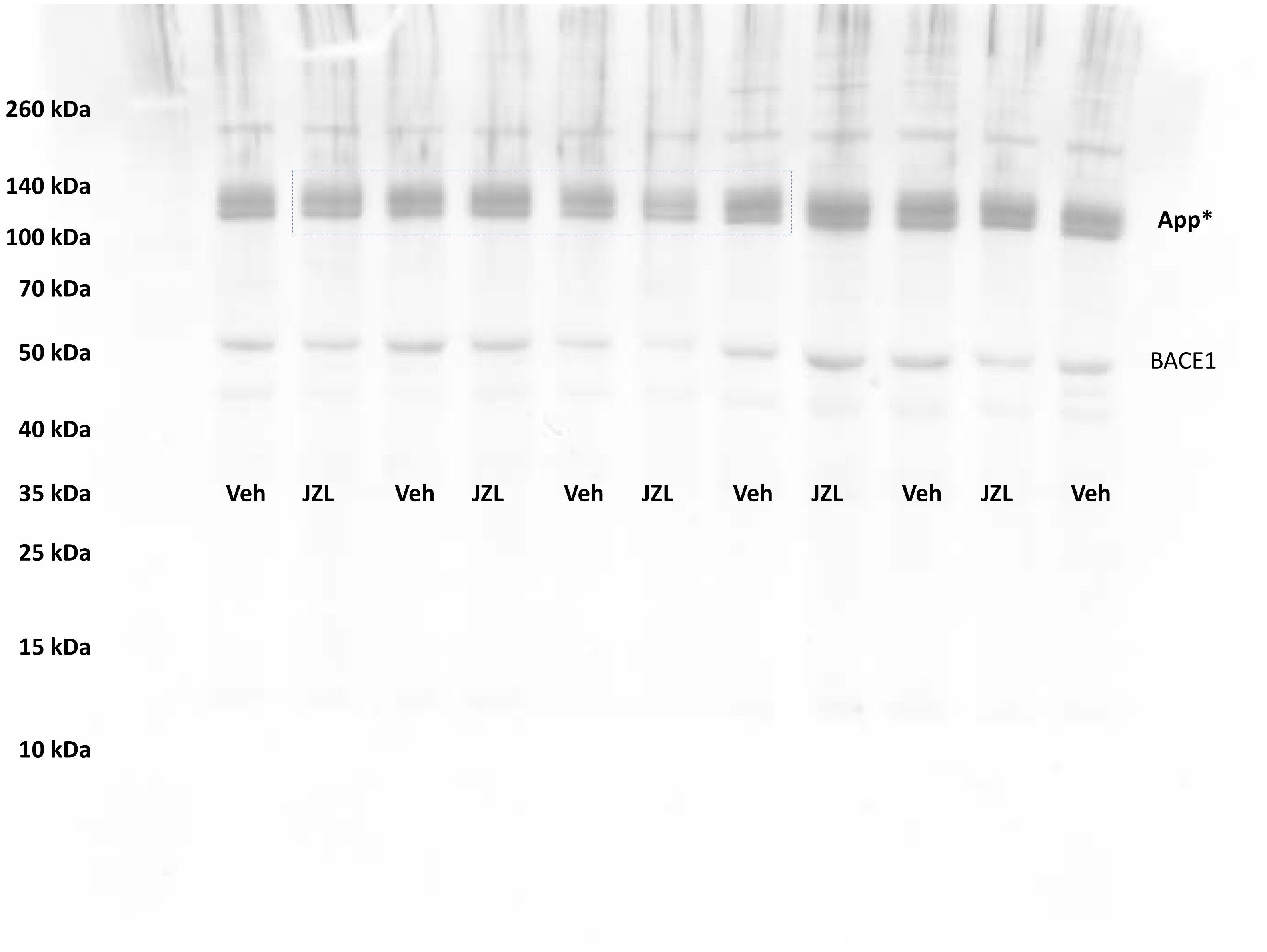

Figure S2H. Blot 3 (Ts Veh vs. Ts JZL) after App + βActin antibodies

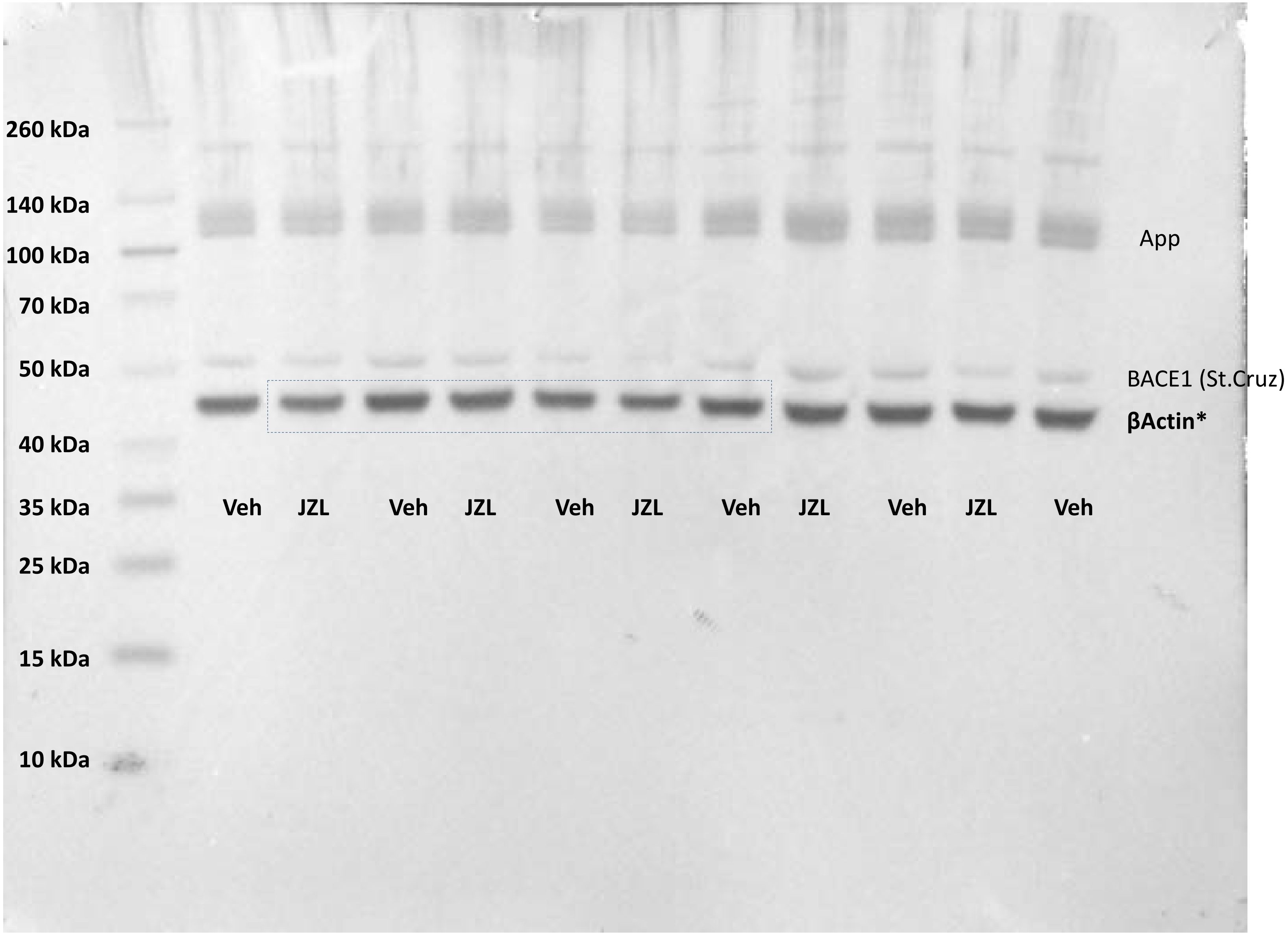

Figure S2I. Blot 3 (Ts Veh vs. Ts JZL) after App+ βActin + BACE1 antibodies

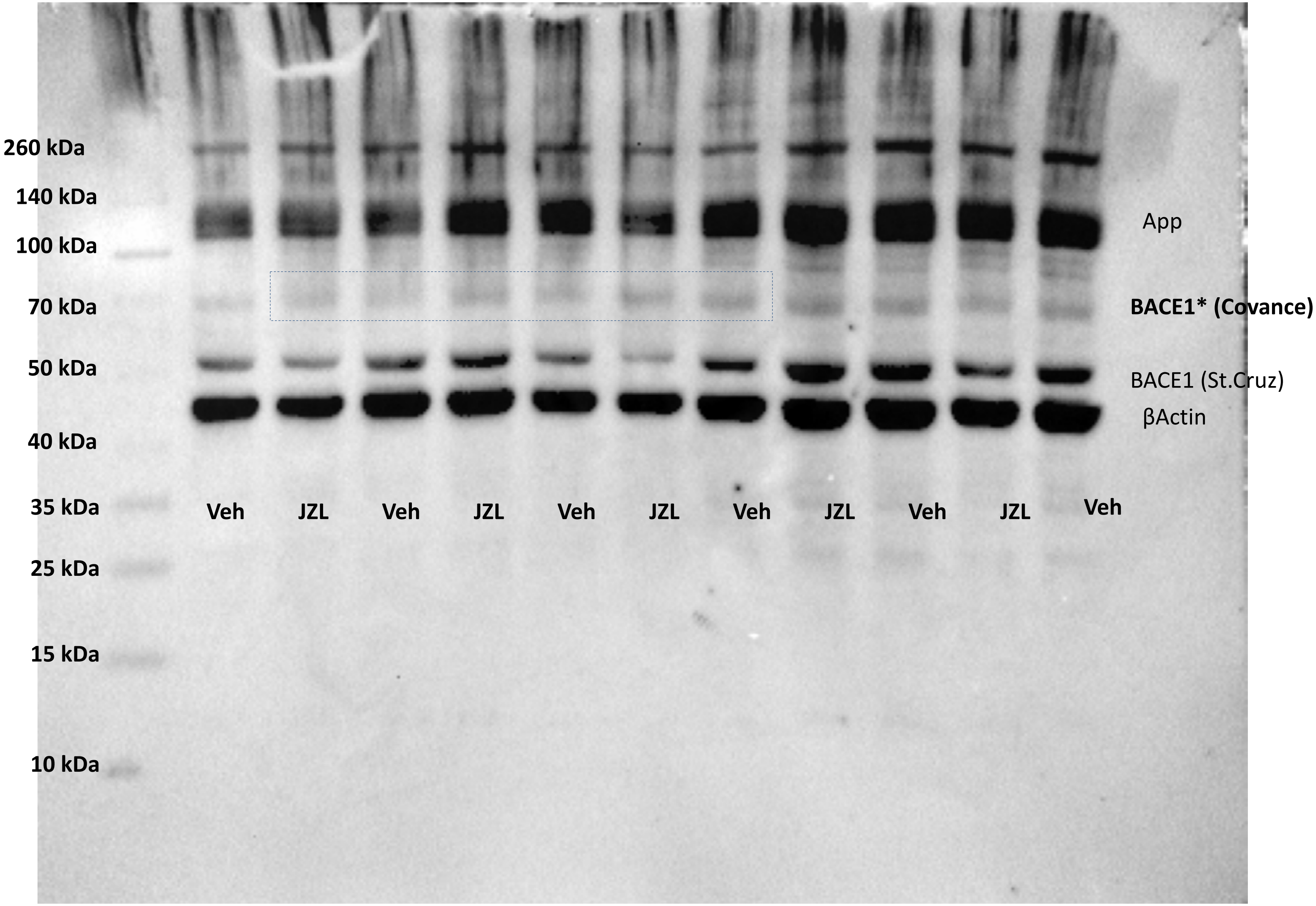

Supplement: Figure S2 — Original Western blots. A. Blot # 1 with alternatively placed samples of the vehicle-treated 2N and Ts65Dn mice. The blot was exposed to the rabbit polyclonal anti-App antibody (the first primary antibody). The part of the figure marked by the dotted rectangle is shown in Figure 7A as ‘App’. In addition, this and other blots were exposed to anti-BACE1 antibody (M-83, Santa Cruz, 1∶1000), which produced a band at ∼51 kDa, i.e. significantly lower than the expected for BACE1 value of ∼70 kDa. Since the correct identify of this band could not be confirmed with a blocking peptide, the results for this band were not include in the paper. Of note, evaluation of results for this band showed no effect of JZL184 treatment in both 2N and Ts65Dn samples, which is similar to the results observed for the 70 kDa band. B. Blot # 1 after exposure to the rabbit anti- βActin antibody (the second primary antibody). Lines for both App and βActin are visible. The part of the figure marked by the dotted rectangle is shown in Figure 7A as ‘βActin’. C. Blot # 1 after exposure to the rabbit anti- BACE1 antibody (the third primary antibody). Lines for BACE1, App, and App are visible. The part of the figure marked by the dotted rectangle is shown in Figure 7A as ‘BACE1’. D. Blot # 2 with alternatively placed samples of the vehicle- and JZL-treated 2N mice. The blot was exposed to the rabbit polyclonal anti-App antibody (the first primary antibody). The part of the figure marked by the dotted rectangle is shown in Figure 7B as ‘App’. E. Blot # 2 after exposure to the rabbit anti-βActin antibody (the second primary antibody). Lines for both App and βActin are visible. The part of the figure marked by the dotted rectangle is shown in Figure 7B as ‘βActin’. F. Blot # 2 after exposure to the rabbit anti-BACE1 antibody (the third primary antibody). Lines for BACE1, App, and App are visible. The part of the figure marked by the dotted rectangle is shown in Figure 7B as ‘BACE1’. G. Blot # 3 with alte [file pone.0114521.s002.pdf]
