# Supplementary material for: Monoacylglycerol Lipase Inhibitor JZL184 Improves Behavior and Neural Properties in Ts65Dn Mice, a Model of Down Syndrome
Source: PLoS One. 2014 Dec 4;9(12):e114521. doi: 10.1371/journal.pone.0114521 (PMC4256450; doi:10.1371/journal.pone.0114521)
Supplement: Table S1 — Effect of chronic JZL184 treatment on the brain levels of lipids in Ts65Dn vs. 2N mice. Targeted metabolomic measurements were performed via multiple-reaction monitoring (MRM) mass spectrometry on lipid extracts from the brains of Ts65Dn and 2N mice treated with vehicle or JZL184. Metabolite levels are shown relative to the brains from 2N vehicle-treated mice. Two-way ANOVA F ratios and p values, as well as p-values for Student's two-tails T-test are shown and values of statistical significance (p<0.05) are shown in bold. (DOCX) [file pone.0114521.s003.docx]

**Supplemental Table S1. Effect of chronic JZL184 treatment on the brain levels of lipids in Ts65Dn vs. 2N mice**

**Negative ionization mode:**

| **Lipid species** | **2N Veh**  **(n=8)** | **2N JZL184**  **(n=8)** | **Ts Veh**  **(n=7)** | **Ts JZL184 (n=7)** | **ANOVA** | **Factor 1:**  **Genotype** | **Factor 2:**  **Treatment** | **Interaction**  **Gen x Treat** | **p**  **2N Veh vs. Ts Veh** | **p**  **2N Veh vs. 2N JZL** | **p**  **Ts Veh vs. Ts JZL** | **p**  **2N Veh vs. Ts JZL** |
| --- | --- | --- | --- | --- | --- | --- | --- | --- | --- | --- | --- | --- |
| Acyl DHAP | 100 ± 38.7 | 67.4 ± 20.0 | 83.5 ± 29.0 | 78.6 ± 29.9 | F_1,26_  p | 0.01  0.93 | 0.44  0.51 | 0.24  0.63 | 0.73 | 0.44 | 0.90 | 0.65 |
| Alkyl glycerone phosphate C16:0 | 100 ± 21.6 | 87.8 ± 12.3 | 127.5 ± 38.9 | 125.1 ± 21.0 | F_1,26_  p | 2.02  0.17 | 0.10  0.75 | 0.05  0.83 | 0.50 | 0.61 | 0.95 | 0.39 |
| Alkyl LPA C16:0 | 100 ± 11.0 | 93.1 ± 10.6 | 127.4 ± 12.0 | 114.3 ± 23.4 | F_1,26_  p | 3.15  0.09 | 0.53  0.47 | 0.05  0.82 | 0.09 | 0.64 | 0.60 | 0.54 |
| Alkyl LPA C18:0 | 100 ± 19.1 | 69.7 ± 7.3 | 109.5 ± 17.7 | 92.7 ± 18.0 | F_1,26_  p | 1.18  0.29 | 2.48  0.13 | 0.20  0.66 | 0.70 | 0.14 | 0.49 | 0.77 |
| Alkyl LPA C18:1 | 100 ± 14.7 | 78.6 ± 6.7 | 116.5 ± 13.4 | 99.0 ± 15.3 | F_1,26_  p | 2.39  0.13 | 2.65  0.12 | 0.03  0.87 | 0.39 | 0.18 | 0.37 | 0.96 |
| Alkyl LPI C16:0 | 100 ± 16.7 | 65.1 ± 7.9 | 96.4 ± 16.6 | 84.1 ± 13.3 | F_1,26_  p | 0.35  0.56 | 3.27  0.08 | 0.75  0.39 | 0.87 | 0.06 | 0.54 | 0.45 |
| Alkyl LPI C18:0 | 100 ± 15.9 | 109.2 ± 20.6 | 106.6 ± 30.6 | 89.5 ± 13.9 | F_1,26_  p | 0.11  0.74 | 0.04  0.84 | 0.46  0.51 | 0.83 | 0.71 | 0.59 | 0.61 |
| Alkyl LPI C18:1 | 100 ± 11.9 | 91.8 ± 13.4 | 73.9 ± 13.9 | 88.1 ± 11.5 | F_1,26_  p | 1.57  0.22 | 0.06  0.81 | 0.88  0.36 | 0.15 | 0.63 | 0.41 | 0.46 |
| Alkyl LPI C20:4 | 100 ± 15.0 | 111.4 ± 36.5 | 74.5 ± 15.5 | 80.0 ± 23.7 | F_1,26_  p | 1.49  0.23 | 0.13  0.72 | 0.02  0.90 | 0.23 | 0.76 | 0.84 | 0.44 |
| **Alkyl PA C16:0/18:1** | **100 ± 15.5** | **48.1 ± 8.4** | **110.5 ± 23.1** | **87.3 ± 19.2** | **F_1,26_**  **p** | 2.51  0.13 | **5.72**  **0.02** | 0.84  0.37 | 0.69 | **0.01** | 0.42 | 0.59 |
| Alkyl PA C16:0/20:4 | 100 ± 16.9 | 65.0 ± 10.8 | 127.1 ± 31.3 | 89.5 ± 25.3 | F_1,26_  p | 1.65  0.21 | 3.27  0.08 | 4.12E-03  0.95 | 0.41 | 0.08 | 0.33 | 0.71 |
| Alkyl PA C18:0/C18:1 | 100 ± 26.4 | 93.0 ± 13.5 | 150.4 ± 31.7 | 106.4 ± 19.0 | F_1,26_  p | 2.14  0.16 | 1.37  0.25 | 0.72  0.40 | 0.21 | 0.81 | 0.22 | 0.84 |
| **Alkyl PA C18:0/C20:4** | **100 ± 11.9** | **76.0 ± 7.4** | **116.2 ± 16.3** | **81.8 ± 11.5** | **F_1,26_**  **p** | 0.99  0.33 | **6.94**  **0.01** | 0.22  0.64 | 0.40 | 0.09 | 0.09 | 0.26 |
| Alkyl PI C16:0/18:1 | 100 ± 6.2 | 82.9 ± 5.9 | 82.7 ± 12.1 | 96.1 ± 12.0 | F_1,26_  p | 0.06  0.81 | 0.05  0.83 | 3.20  0.09 | 0.18 | 0.05 | 0.41 | 0.75 |
| Alkyl PI C16:0/20:4 | 100 ± 4.5 | 95.5 ± 6.4 | 89.1 ± 7.7 | 95.0 ± 15.9 | F_1,26_  p | 0.44  0.51 | 0.01  0.94 | 0.37  0.55 | 0.20 | 0.55 | 0.72 | 0.73 |
| Alkyl PI C18:0/C18:1 | 100 ± 9.4 | 87.3 ± 14.2 | 93.9 ± 6.5 | 87.5 ± 13.4 | F_1,26_  p | 0.07  0.79 | 0.80  0.38 | 0.09  0.77 | 0.59 | 0.44 | 0.65 | 0.42 |
| Alkyl PI C18:0/C20:4 | 100 ± 8.5 | 92.3 ± 4.7 | 94.4 ± 12.3 | 101.9 ± 7.2 | F_1,26_  p | 0.07  0.80 | 3.11E-04  0.99 | 0.94  0.34 | 0.69 | 0.41 | 0.58 | 0.86 |
| **DHA** | **100 ± 9.4** | **62.3 ± 7.1** | **97.6 ± 9.0** | **76.1 ± 12.2** | **F_1,26_**  **p** | 0.42  0.52 | **11.32**  **2.39E-03** | 0.85  0.36 | 0.85 | 0.00 | 0.15 | 0.11 |
| EPA | 100 ± 14.8 | 90.0 ± 14.1 | 104.2 ± 19.5 | 181.7 ± 67.1 | F_1,26_  p | 2.27  0.14 | 1.13  0.30 | 1.89  0.18 | 0.85 | 0.61 | 0.25 | 0.19 |
| FFA C16:0 | 100 ± 10.7 | 86.5 ± 12.6 | 118.6 ± 8.7 | 116.8 ± 19.3 | F_1,26_  p | 3.93  0.06 | 0.39  0.54 | 0.22  0.64 | 0.18 | 0.40 | 0.93 | 0.41 |
| FFA C18:0 | 100 ± 9.5 | 86.2 ± 9.3 | 107.6 ± 7.6 | 99.2 ± 12.1 | F_1,26_  p | 1.28  0.27 | 1.49  0.23 | 0.09  0.77 | 0.52 | 0.29 | 0.54 | 0.96 |
| FFA C18:1 | 100 ± 11.9 | 84.3 ± 13.8 | 116.1 ± 12.3 | 99.1 ± 13.9 | F_1,26_  p | 1.61  0.22 | 1.79  0.19 | 2.81E-03  0.96 | 0.33 | 0.38 | 0.34 | 0.96 |
| **FFA C20:4 (AA)** | **100 ± 9.1** | **30.2 ± 2.0** | **90.9 ± 5.9** | **27.4 ± 3.1** | **F_1,26_**  **p** | 1.17  0.29 | **147.30**  **3.26E-12** | 0.34  0.57 | **0.40** | **1.37E-06** | **2.54E-07** | **3.85E-06** |
| Glucosyl-ceramide | 100 ± 19.2 | 82.6 ± 17.2 | 114.5 ± 22.3 | 135.0 ± 37.4 | F_1,26_  p | 2.15  0.15 | 4.70E-03  0.95 | 0.69  0.41 | 0.60 | 0.48 | 0.62 | 0.37 |
| LPA C16:0 | 100 ± 18.9 | 80.4 ± 13.0 | 181.0 ± 85.9 | 100.5 ± 17.1 | F_1,26_  p | 1.65  0.21 | 1.61  0.22 | 0.60  0.45 | 0.31 | 0.38 | 0.34 | 0.98 |
| LPA C18:0 | 100 ± 11.4 | 86.0 ± 3.9 | 115.9 ± 11.8 | 107.5 ± 14.7 | F_1,26_  p | 3.41  0.08 | 1.22  0.28 | 0.08  0.78 | 0.32 | 0.23 | 0.64 | 0.67 |
| LPA C18:1 | 100 ± 10.5 | 82.3 ± 9.9 | 113.5 ± 9.7 | 107.2 ± 17.1 | F_1,26_  p | 2.96  0.10 | 1.16  0.29 | 0.26  0.62 | 0.33 | 0.21 | 0.73 | 0.70 |
| LPI C16:0 | 100 ± 12.2 | 88.1 ± 7.9 | 117.1 ± 14.3 | 105.8 ± 15.1 | F_1,26_  p | 2.26  0.14 | 1.01  0.32 | 0.00  0.98 | 0.34 | 0.40 | 0.57 | 0.75 |
| LPI C18:0 | 100 ± 11.8 | 78.1 ± 7.4 | 109.9 ± 14.3 | 89.6 ± 12.0 | F_1,26_  p | 1.00  0.33 | 3.91  0.06 | 0.01  0.94 | 0.57 | 0.12 | 0.26 | 0.52 |
| LPI C18:1 | 100 ± 13.0 | 80.7 ± 8.2 | 113.3 ± 17.8 | 91.5 ± 14.2 | F_1,26_  p | 0.94  0.34 | 2.72  0.11 | 0.01  0.92 | 0.52 | 0.20 | 0.32 | 0.64 |
| LPI C20:4 | 100 ± 16.7 | 84.2 ± 15.0 | 122.0 ± 16.0 | 98.4 ± 17.9 | F_1,26_  p | 1.41  0.25 | 1.66  0.21 | 0.06  0.80 | 0.33 | 0.46 | 0.31 | 0.95 |
| **PA C16:0/C18:1** | **100 ± 9.7** | **78.3 ± 7.0** | **103.7 ± 17.8** | **76.7 ± 11.4** | **F_1,26_**  **p** | 0.01  0.92 | **5.00**  **0.03** | 0.06  0.81 | 0.84 | 0.07 | 0.19 | 0.12 |
| **PA C16:0/C20:4** | **100 ± 12.7** | **77.7 ± 9.0** | **102.7 ± 16.9** | **68.4 ± 17.8** | **F_1,26_**  **p** | 0.06  0.80 | **4.62**  **0.04** | 0.21  0.65 | 0.89 | 0.15 | 0.16 | 0.14 |
| **PA C18:0/C18:1** | **100 ± 8.2** | **81.8 ± 4.5** | **107.9 ± 9.8** | **109.7 ± 6.5** | **F_1,26_**  **p** | **6.74**  **0.02** | 1.41  0.25 | 2.11  0.16 | 0.52 | 0.05 | 0.87 | 0.35 |
| PA C18:0/C20:4 | 100 ± 16.6 | 78.6 ± 10.1 | 110.2 ± 16.3 | 96.2 ± 21.4 | F_1,26_  p | 0.84  0.37 | 1.36  0.25 | 0.06  0.81 | 0.65 | 0.26 | 0.59 | 0.88 |
| **PGD2^a^** | **100 ± 10.6** | **22.6 ± 2.8** | **89.0 ± 6.4** | **17.4 ± 2.1** | **F_1,26_**  **p** | 1.70  0.20 | **144.23**  **4.12E-12** | 0.21  0.65 | **0.38** | **2.61E-06** | **7.33E-08** | **3.59E-06** |
| **PGE2 ^a^** | **100 ± 16.2** | **28.6 ± 4.2** | **84.8 ± 9.2** | **34.2 ± 5.6** | **F_1,26_**  **p** | 0.25  0.62 | **40.56**  **9.58E-07** | 1.18  0.29 | **0.41** | **4.36E-04** | **2.65E-04** | **1.87E-03** |
| **PGF2α ^a^** | **100 ± 14.8** | **26.8 ± 4.3** | **84.6 ± 8.7** | **36.2 ± 7.9** | **F_1,26_**  **p** | 0.10  0.75 | **43.23**  **5.68E-07** | 1.80  0.19 | **0.37** | **1.70E-04** | **7.73E-04** | **1.84E-03** |
| **PGJ2 ^a^** | **100 ± 13.8** | **33.3 ± 4.9** | **88.0 ± 4.8** | **32.5 ± 4.3** | **F_1,26_**  **p** | 0.67  0.42 | **60.29**  **3.08E-08** | 0.51  0.48 | **0.42** | **2.48E-04** | **8.18E-07** | **4.09E-04** |
| PI C16:0/C16:0 | 100 ± 14.1 | 86.3 ± 9.9 | 135.8 ± 21.0 | 107.4 ± 18.9 | F_1,26_  p | 3.63  0.07 | 1.99  0.17 | 0.24  0.63 | 0.14 | 0.41 | 0.30 | 0.74 |
| PI C16:0/C18:1 | 100 ± 6.6 | 93.1 ± 4.9 | 119.8 ± 13.2 | 108.0 ± 11.9 | F_1,26_  p | 3.98  0.06 | 1.15  0.29 | 0.08  0.78 | 0.16 | 0.39 | 0.49 | 0.52 |
| **PI C16:0/C20:4** | **100 ± 11.9** | **73.8 ± 10.1** | **127.2 ± 21.9** | **95.9 ± 16.5** | **F_1,26_**  **p** | 3.02  0.09 | **4.11**  **0.05** | 0.03  0.86 | 0.24 | 0.09 | 0.24 | 0.83 |
| PI C18:0/C18:1 | 100 ± 6.1 | 97.6 ± 7.4 | 105.2 ± 6.5 | 106.7± 4.9 | F_1,26_  p | 1.44  0.24 | 4.86E-03  0.94 | 0.11  0.75 | 0.54 | 0.80 | 0.84 | 0.38 |
| PI C18:0/C20:4 | 100 ± 5.5 | 86.9 ± 4.5 | 105.5 ± 9.5 | 97.7 ± 5.5 | F_1,26_  p | 1.89  0.18 | 3.10  0.09 | 0.20  0.66 | 0.59 | 0.07 | 0.46 | 0.76 |
| Sphingosine phosphate C16:0 | 100 ± 18.3 | 82.3 ± 7.2 | 101.5 ± 12.3 | 91.2 ± 12.8 | F_1,26_  p | 0.17  0.68 | 1.26  0.27 | 0.09  0.77 | 0.94 | 0.35 | 0.54 | 0.69 |
| **Txb2** | **100 ± 13.9** | **45.0 ± 5.1** | **106.7 ± 12.2** | **71.9± 17.8** | **F_1,26_**  **p** | 2.01  0.17 | **14.29**  **8.28E-04** | 0.72  0.40 | **0.71** | **1.36E-03** | **0.11** | **0.20** |

**Positive ionization mode:**

| **Lipid species** | **2N Veh**  **(n=8)** | **2N JZL184**  **(n=8)** | **Ts Veh**  **(n=7)** | **Ts JZL184 (n=7)** | **ANOVA** | **ANOVA**  **Factor 1:**  **Genotype** | **ANOVA**  **Factor 2:**  **Treatment** | **ANOVA**  **Interaction**  **Gen x Treat** | **p**  **2N Veh vs Ts Veh** | **p**  **2N Veh vs. 2N JZL** | **p**  **Ts Veh vs. Ts JZL** | **p**  **2N Veh vs. Ts JZL** |
| --- | --- | --- | --- | --- | --- | --- | --- | --- | --- | --- | --- | --- |
| AcMAGE C16:0 | 100 ± 11.8 | 93.8 ± 12.4 | 102.9 ± 19.5 | 105.8 ± 8.4 | F_1,26_  p | 0.35  0.56 | 0.02  0.89 | 0.13  0.72 | 0.89 | 0.70 | 0.89 | 0.68 |
| AcMAGE C18:0 | 100 ± 11.7 | 92.3 ± 15.2 | 120.3 ± 18.5 | 124.1 ± 24.1 | F_1,26_  p | 2.57  0.12 | 0.01  0.91 | 0.13  0.73 | 0.33 | 0.67 | 0.89 | 0.33 |
| AcMAGE C18:1 | 100 ± 6.6 | 98.0 ± 7.0 | 101.4 ± 10.9 | 104.4 ± 13.8 | F_1,26_  p | 0.19  0.67 | 0.00  0.96 | 0.08  0.78 | 0.90 | 0.83 | 0.86 | 0.75 |
| acyl carnitine C02:0 | 100 ± 13.4 | 84.2 ± 5.8 | 123.8 ± 14.7 | 112.4 ± 23.5 | F_1,26_  p | 3.45  0.07 | 0.94  0.34 | 0.03  0.87 | 0.22 | 0.27 | 0.67 | 0.62 |
| acyl carnitine C04:0 | 100 ± 24.1 | 70.6 ± 5.8 | 91.4 ± 17.9 | 92.4 ± 20.9 | F_1,26_  p | 0.15  0.70 | 0.68  0.42 | 0.78  0.38 | 0.77 | 0.23 | 0.97 | 0.81 |
| acyl carnitine C06:0 | 100 ± 19.1 | 80.5 ± 16.5 | 89.7 ± 16.0 | 99.3 ± 26.1 | F_1,26_  p | 0.05  0.82 | 0.07  0.79 | 0.64  0.43 | 0.67 | 0.42 | 0.74 | 0.98 |
| acyl carnitine C12:0 | 100 ± 15.2 | 83.7 ± 16.0 | 133.1 ± 20.2 | 118.9 ± 35.5 | F_1,26_  p | 2.69  0.11 | 0.54  0.47 | 2.58E-03  0.96 | 0.18 | 0.44 | 0.71 | 0.59 |
| acyl carnitine C16:0 | 100 ± 12.7 | 82.2 ± 14.9 | 134.5 ± 23.8 | 128.0 ± 33.2 | F_1,26_  p | 3.96  0.06 | 0.36  0.55 | 0.08  0.78 | 0.18 | 0.35 | 0.87 | 0.39 |
| **acyl carnitine C18:0** | **100 ± 16.0** | **76.4 ± 11.6** | **139.5 ± 26.9** | **124.9 ± 33.9** | **F_1,26_**  **p** | **4.34**  **0.05** | 0.82  0.37 | 0.04  0.83 | 0.18 | 0.22 | 0.72 | 0.47 |
| alkyl LPC C16:0 | 100 ± 33.8 | 106.8 ± 24.3 | 128.5 ± 22.4 | 141.4 ± 41.1 | F_1,26_  p | 1.18  0.29 | 0.12  0.74 | 0.01  0.92 | 0.48 | 0.86 | 0.77 | 0.41 |
| alkyl LPC C18:0 | 100 ± 24.3 | 72.8 ± 19.6 | 143.9 ± 25.5 | 106.5 ± 41.1 | F_1,26_  p | 2.21  0.15 | 1.53  0.23 | 0.04  0.85 | 0.20 | 0.37 | 0.42 | 0.88 |
| alkyl LPC C18:1 | 100 ± 18.4 | 70.2 ± 25.8 | 102.4 ± 32.5 | 98.3 ± 25.1 | F_1,26_  p | 0.41  0.53 | 0.51  0.48 | 0.29  0.59 | 0.94 | 0.33 | 0.92 | 0.95 |
| alkyl LPE C16:0 | 100 ± 12.2 | 117.7 ± 12.7 | 116.4 ± 8.5 | 129.1 ± 12.4 | F_1,26_  p | 1.61  0.22 | 1.92  0.18 | 0.05  0.82 | 0.27 | 0.30 | 0.38 | 0.10 |
| alkyl LPE C18:0 | 100 ± 16.7 | 81.4 ± 13.0 | 120.0 ± 13.4 | 106.7 ± 29.4 | F_1,26_  p | 1.68  0.21 | 0.83  0.37 | 0.02  0.88 | 0.34 | 0.36 | 0.66 | 0.83 |
| alkyl LPE C18:1 | 100 ± 17.3 | 76.7 ± 13.3 | 126.8 ± 20.1 | 106.7 ± 30.4 | F_1,26_  p | 2.19  0.15 | 1.29  0.27 | 0.01  0.94 | 0.29 | 0.27 | 0.56 | 0.84 |
| alkyl LPE C20:4 | 100 ± 4.8 | 98.9 ± 3.4 | 100.4 ± 10.5 | 108.7 ± 10.9 | F_1,26_  p | 0.51  0.48 | 0.26  0.62 | 0.44  0.51 | 0.97 | 0.85 | 0.56 | 0.42 |
| alkyl LPG C16:0 | 100 ± 22.2 | 174.2 ± 66.4 | 259.3 ± 71.3 | 141.2 ± 38.0 | F_1,26_  p | 1.55  0.22 | 0.19  0.67 | 3.60  0.07 | **0.03** | 0.28 | 0.16 | 0.30 |
| alkyl LPG C18:0 | 100 ± 35.0 | 118.9 ± 34.4 | 67.2 ± 10.0 | 78.2 ± 13.4 | F_1,26_  p | 2.05  0.16 | 0.34  0.57 | 0.02  0.88 | 0.38 | 0.69 | 0.49 | 0.56 |
| alkyl LPS C18:0 | 100 ± 8.4 | 100.0 ± 22.4 | 110.8 ± 15.4 | 134.4 ± 24.2 | F_1,26_  p | 1.72  0.20 | 0.47  0.50 | 0.47  0.50 | 0.51 | 1.00 | 0.39 | 0.15 |
| alkyl LPS C18:1 | 100 ± 29.0 | 84.5 ± 22.3 | 93.5 ± 32.9 | 105.4 ± 32.9 | F_1,26_  p | 0.07  0.79 | 0.00  0.95 | 0.25  0.62 | 0.88 | 0.66 | 0.79 | 0.90 |
| alkyl PC C16:0/18:1 | 100 ± 14.8 | 97.9 ± 18.0 | 99.8 ± 10.5 | 97.3 ± 13.4 | F_1,26_  p | 7.06E-04  0.98 | 0.03  0.87 | 1.68E-04  0.99 | 0.99 | 0.92 | 0.88 | 0.89 |
| alkyl PC C16:0/20:4 | 100 ± 5.8 | 99.4 ± 5.5 | 88.3 ± 6.2 | 102.7 ± 7.7 | F_1,26_  p | 0.51  0.48 | 1.37  0.25 | 1.63  0.21 | 0.16 | 0.93 | 0.14 | 0.77 |
| alkyl PC C18:0/C18:1 | 100 ± 10.6 | 88.4 ± 9.4 | 101.8 ± 8.2 | 103.5 ± 11.4 | F_1,26_  p | 0.82  0.37 | 0.28  0.60 | 0.50  0.49 | 0.89 | 0.40 | 0.90 | 0.81 |
| alkyl PC C18:0/C20:4 | 100 ± 3.6 | 92.0 ± 5.0 | 89.2 ± 7.8 | 100.5 ± 9.1 | F_1,26_  p | 0.04  0.85 | 0.07  0.79 | 2.58  0.12 | 0.18 | 0.19 | 0.33 | 0.95 |
| **alkyl PE C16:0/18:1** | **100 ± 7.5** | **81.8 ± 6.2** | **82.4 ± 7.9** | **92.6 ± 6.4** | F_1,26_  p | 0.27  0.61 | 0.37  0.55 | **4.71**  **0.04** | 0.11 | 0.06 | 0.30 | 0.44 |
| alkyl PE C16:0/20:4 | 100 ± 6.9 | 89.5 ± 6.6 | 83.9 ± 7.5 | 97.8 ± 5.8 | F_1,26_  p | 0.38  0.54 | 0.07  0.79 | 3.74  0.06 | 0.11 | 0.26 | 0.14 | 0.80 |
| **alkyl PE C18:0/C18:1** | **100 ± 6.4** | **81.2 ± 5.6** | **88.3 ± 5.6** | **92.2 ± 5.1** | F_1,26_  p | 0.00  0.95 | 1.91  0.18 | **4.46**  **0.04** | 0.17 | 0.03 | 0.59 | 0.34 |
| alkyl PE C18:0/C20:4 | 100 ± 34.2 | 93.0 ± 30.2 | 126.0 ± 30.9 | 108.1 ± 33.7 | F_1,26_  p | 0.46  0.50 | 0.17  0.68 | 0.03  0.86 | 0.56 | 0.87 | 0.68 | 0.86 |
| alkyl PG C16:0/18:1 | 100 ± 10.5 | 91.6 ± 10.7 | 86.0 ± 5.9 | 109.6 ± 14.2 | F_1,26_  p | 0.04  0.85 | 0.58  0.45 | 2.53  0.12 | 0.25 | 0.56 | 0.12 | 0.57 |
| alkyl PG C16:0/20:4 | 100 ± 6.4 | 88.1 ± 8.3 | 76.8 ± 9.4 | 93.6 ± 9.2 | F_1,26_  p | 1.32  0.26 | 0.10  0.75 | 3.43  0.08 | **0.04** | 0.25 | 0.19 | 0.54 |
| **alkyl PG C18:0/C18:1** | **100 ± 3.6** | **83.4 ± 8.7** | **85.3 ± 9.0** | **97.3 ± 6.2** | F_1,26_  p | 2.74E-03  0.96 | 0.12  0.73 | **4.59**  **0.04** | 0.11 | 0.08 | 0.26 | 0.69 |
| alkyl PG C18:0/C20:4 | 100 ± 22.1 | 98.3 ± 17.2 | 113.2 ± 10.4 | 141.6 ± 16.3 | F_1,26_  p | 2.98  0.10 | 0.67  0.42 | 0.85  0.36 | 0.59 | 0.95 | 0.14 | 0.14 |
| alkyl PS C16:0/18:1 | 100 ± 6.7 | 78.9 ± 9.1 | 97.3 ± 6.8 | 101.6 ± 9.1 | F_1,26_  p | 1.77  0.19 | 1.26  0.27 | 2.86  0.10 | 0.77 | 0.07 | 0.69 | 0.88 |
| alkyl PS C16:0/20:4 | 100 ± 7.0 | 114.2 ± 21.6 | 107.4 ± 12.3 | 133.1 ± 20.6 | F_1,26_  p | 0.73  0.40 | 1.69  0.21 | 0.14  0.71 | 0.57 | 0.51 | 0.27 | 0.11 |
| **alkyl PS C18:0/C18:1** | **100 ± 9.0** | **82.4 ± 6.3** | **87.1 ± 8.2** | **100.6 ± 7.8** | **F_1,26_**  **p** | 0.14  0.72 | 0.08  0.78 | **4.48**  **0.04** | 0.28 | 0.11 | 0.22 | 0.96 |
| alkyl PS C18:0/C20:4 | 100 ± 8.0 | 93.3 ± 8.4 | 98.2 ± 12.5 | 109.5 ± 8.9 | F_1,26_  p | 0.67  0.42 | 0.07  0.80 | 1.04  0.32 | 0.90 | 0.55 | 0.44 | 0.41 |
| Ceramide C02:0 | 100 ± 25.7 | 139.1 ± 35.6 | 96.5 ± 27.3 | 178.4 ± 54.4 | F_1,26_  p | 0.27  0.61 | 3.12  0.09 | 0.39  0.54 | 0.92 | 0.36 | 0.17 | 0.17 |
| Ceramide C16:0 | 100 ± 22.3 | 79.1 ± 27.1 | 121.2 ± 10.6 | 111.3 ± 29.9 | F_1,26_  p | 1.43  0.24 | 0.48  0.49 | 0.06  0.81 | 0.40 | 0.53 | 0.74 | 0.75 |
| Ceramide C18:0 | 100 ± 20.2 | 72.6 ± 19.1 | 101.4 ± 6.6 | 111.2 ± 28.4 | F_1,26_  p | 1.14  0.30 | 0.22  0.64 | 0.98  0.33 | 0.95 | 0.31 | 0.72 | 0.73 |
| Ceramide C18:1 | 100 ± 9.1 | 89.1 ± 10.3 | 92.5 ± 8.7 | 88.2 ± 10.4 | F_1,26_  p | 0.22  0.64 | 0.71  0.41 | 0.14  0.72 | 0.53 | 0.41 | 0.74 | 0.37 |
| Ceramide C20:4 | 100 ± 10.2 | 90.5 ± 11.7 | 95.6 ± 4.9 | 133.3 ± 24.0 | F_1,26_  p | 2.15  0.15 | 1.15  0.29 | 3.24  0.08 | 0.70 | 0.52 | 0.12 | 0.17 |
| DAG C16:0/C18:1 | 100 ± 20.8 | 88.0 ± 21.1 | 119.5 ± 9.6 | 111.9 ± 23.2 | F_1,26_  p | 1.39  0.25 | 0.29  0.60 | 0.01  0.91 | 0.40 | 0.67 | 0.75 | 0.69 |
| DAG C16:0/C20:4 | 100 ± 22.5 | 102.4 ± 16.3 | 122.6 ± 25.4 | 135.0 ± 15.7 | F_1,26_  p | 2.12  0.16 | 0.15  0.70 | 0.07  0.79 | 0.49 | 0.93 | 0.66 | 0.21 |
| DAG C18:0/C18:1 | 100 ± 20.2 | 93.2 ± 23.3 | 129.4 ± 13.4 | 121.0 ± 26.1 | F_1,26_  p | 2.04  0.16 | 0.14  0.71 | 1.68E-03  0.97 | 0.23 | 0.82 | 0.76 | 0.50 |
| **DAG C18:0/C20:4** | **100 ± 10.4** | **85.9 ± 14.0** | **71.6 ± 12.4** | **183.6 ± 67.8** | F_1,26_  p | 1.25  0.27 | 2.50  0.13 | **4.15**  **0.05** | 0.08 | 0.40 | 0.10 | 0.18 |
| LPC C16:0 | 100 ± 22.0 | 72.7 ± 24.3 | 139.8 ± 21.8 | 107.7 ± 34.9 | F_1,26_  p | 2.39  0.13 | 1.50  0.23 | 0.01  0.92 | 0.19 | 0.39 | 0.42 | 0.84 |
| LPC C18:0 | 100 ± 30.5 | 80.4 ± 23.8 | 138.8 ± 30.8 | 119.1 ± 29.3 | F_1,26_  p | 2.11  0.16 | 0.54  0.47 | 1.93E-06  1.00 | 0.36 | 0.60 | 0.63 | 0.64 |
| LPC C18:1 | 100 ± 17.5 | 76.8 ± 17.6 | 130.2 ± 19.2 | 111.2 ± 32.3 | F_1,26_  p | 2.49  0.13 | 1.06  0.31 | 0.01  0.92 | 0.23 | 0.34 | 0.60 | 0.74 |
| LPC C20:4 | 100 ± 23.1 | 72.1 ± 25.9 | 133.7 ± 29.6 | 116.1 ± 37.7 | F_1,26_  p | 2.06  0.16 | 0.71  0.41 | 0.04  0.85 | 0.35 | 0.40 | 0.70 | 0.69 |
| LPE C16:0 | 100 ± 13.8 | 80.5 ± 11.5 | 127.8 ± 15.7 | 106.6 ± 23.2 | F_1,26_  p | 3.18  0.09 | 1.82  0.19 | 3.19E-03  0.96 | 0.18 | 0.27 | 0.43 | 0.79 |
| LPE C18:0 | 100 ± 13.6 | 85.6 ± 13.1 | 130.5 ± 15.8 | 112.4 ± 24.6 | F_1,26_  p | 3.31  0.08 | 1.06  0.31 | 0.01  0.91 | 0.14 | 0.43 | 0.52 | 0.63 |
| LPE C18:1 | 100 ± 16.9 | 75.1 ± 12.5 | 130.6 ± 18.4 | 117.7 ± 33.8 | F_1,26_  p | 3.48  0.07 | 0.93  0.34 | 0.09  0.76 | 0.21 | 0.23 | 0.72 | 0.61 |
| LPE C20:4 | 100 ± 16.0 | 85.0 ± 17.5 | 139.2 ± 20.4 | 122.2 ± 34.9 | F_1,26_  p | 3.28  0.08 | 0.58  0.45 | 2.15E-03  0.96 | 0.12 | 0.51 | 0.66 | 0.53 |
| LPG C18:0 | 100 ± 18.2 | 97.0 ± 16.0 | 168.4 ± 71.1 | 91.4 ± 24.9 | F_1,26_  p | 0.82  0.37 | 1.33  0.26 | 1.14  0.30 | 0.30 | 0.90 | 0.29 | 0.76 |
| LPG C18:1 | 100 ± 20.2 | 192.0 ± 71.8 | 68.6 ± 35.6 | 77.9 ± 22.2 | F_1,26_  p | 3.06  0.09 | 1.48  0.23 | 0.99  0.33 | 0.41 | 0.21 | 0.81 | 0.44 |
| LPG C20:4 | 100 ± 16.0 | 98.2 ± 34.9 | 181.0 ± 37.0 | 127.0 ± 26.5 | F_1,26_  p | 3.99  0.06 | 1.03  0.32 | 0.90  0.35 | **0.04** | 0.96 | 0.22 | 0.35 |
| LPS C16:0 | 100 ± 22.7 | 108.5 ± 36.8 | 187.4 ± 39.5 | 129.0 ± 45.3 | F_1,26_  p | 2.55  0.12 | 0.55  0.47 | 0.98  0.33 | 0.05 | 0.84 | 0.31 | 0.53 |
| LPS C18:0 | 100 ± 16.4 | 84.2 ± 17.7 | 137.4 ± 20.4 | 118.0 ± 35.9 | F_1,26_  p | 2.73  0.11 | 0.67  0.42 | 0.01  0.93 | 0.14 | 0.50 | ` | 0.62 |
| LPS C18:1 | 100 ± 20.1 | 83.1 ± 26.1 | 157.2 ± 30.0 | 138.9 ± 46.4 | F_1,26_  p | 3.80  0.06 | 0.37  0.55 | 6.44E-04  0.98 | 0.10 | 0.59 | 0.73 | 0.40 |
| LPS C20:4 | 100 ± 3.8 | 91.6 ± 4.0 | 95.9 ± 3.2 | 91.1 ± 4.0 | F_1,26_  p | 0.42  0.52 | 3.45  0.07 | 0.27  0.61 | 0.39 | 0.12 | 0.34 | 0.11 |
| MAG C16:0 | 100 ± 10.6 | 113.6 ± 12.1 | 118.2 ± 20.3 | 135.1 ± 11.5 | F_1,26_  p | 2.37  0.14 | 1.39  0.25 | 0.02  0.90 | 0.39 | 0.38 | 0.45 | 0.03 |
| MAG C18:0 | 100 ± 11.5 | 93.8 ± 12.7 | 102.9 ± 19.7 | 106.1 ± 9.3 | F_1,26_  p | 0.36  0.56 | 0.01  0.91 | 0.14  0.71 | 0.89 | 0.71 | 0.87 | 0.67 |
| MAG C18:1 | 100 ± 14.2 | 120.7 ± 21.1 | 142.2 ± 18.5 | 147.2 ± 25.3 | F_1,26_  p | 3.42  0.08 | 0.48  0.50 | 0.18  0.68 | 0.07 | 0.40 | 0.87 | 0.09 |
| **MAG C18:2** | **100 ± 10.5** | **165.3 ± 20.6** | **128.4 ± 19.0** | **172.6 ± 19.2** | **F_1,26_**  **p** | 1.18  0.29 | **11.12**  **2.58E-03** | 0.41  0.53 | 0.17 | 0.01 | 0.10 | 0.00 |
| **MAG C20:4 (2-AG)** | **100 ± 11.0** | **392.9 ± 47.4** | **148.0 ± 15.8** | **451.0 ± 47.7** | **F_1,26_**  **p** | 2.64  0.12 | **83.19**  **1.39E-09** | 0.02  0.88 | **0.02** | **1.57E-05** | **2.90E-05** | **1.61E-06** |
| **MAG C22.6** | **100 ± 13.2** | **167.2 ± 26.3** | **153.1 ± 21.2** | **194.5 ± 33.9** | **F_1,26_**  **p** | 3.13  0.09 | **5.72**  **0.02** | 0.32  0.58 | **0.03** | **0.03** | 0.29 | **0.01** |
| MAGE C16:0 | 100 ± 7.3 | 83.4 ± 7.8 | 94.5 ± 10.7 | 84.5 ± 12.1 | F_1,26_  p | 0.06  0.80 | 2.31  0.14 | 0.14  0.71 | 0.65 | 0.12 | 0.52 | 0.24 |
| MAGE C18:0 | 100 ± 11.9 | 50.0 ± 21.2 | 90.3 ± 22.3 | 87.0 ± 18.9 | F_1,26_  p | 0.61  0.44 | 2.32  0.14 | 1.78  0.19 | 0.68 | 0.04 | 0.90 | 0.53 |
| **MAGE C18:1** | **100 ± 5.7** | **76.0 ± 10.5** | **99.2 ± 16.2** | **81.8 ± 9.1** | **F_1,26_**  **p** | 0.06  0.80 | **4.28**  **0.05** | 0.11  0.75 | 0.96 | 0.05 | 0.33 | 0.08 |
| NAE C16:0 | 100 ± 8.8 | 122.6 ± 19.4 | 148.0 ± 50.2 | 147.0 ± 30.4 | F_1,26_  p | 1.74  0.20 | 0.15  0.70 | 0.19  0.67 | 0.30 | 0.28 | 0.99 | 0.11 |
| NAE C18:0 | 100 ± 14.1 | 100.9 ± 23.4 | 154.0 ± 60.5 | 118.5 ± 31.8 | F_1,26_  p | 1.22  0.28 | 0.28  0.60 | 0.31  0.58 | 0.34 | 0.97 | 0.59 | 0.56 |
| NAE C20:4 or AEA | 100 ± 15.5 | 112.2 ± 19.2 | 115.7 ± 12.9 | 133.5 ± 21.1 | F_1,26_  p | 0.98  0.33 | 3.26  0.08 | 0.13  0.72 | 0.43 | 0.61 | 0.45 | 0.18 |
| PAF/LPC C16:0 | 100 ± 19.7 | 65.1 ± 17.8 | 108.8 ± 23.4 | 99.6 ± 28.2 | F_1,26_  p | 1.10  0.30 | 1.14  0.30 | 0.38  0.54 | 0.76 | 0.18 | 0.79 | 0.99 |
| PC C16:0/C18:1 | 100 ± 8.3 | 91.0 ± 10.9 | 84.1 ± 3.8 | 96.6 ± 5.5 | F_1,26_  p | 0.48  0.50 | 0.05  0.82 | 2.08  0.16 | 0.10 | 0.49 | 0.07 | 0.73 |
| **PC C16:0/C20:4** | **100 ± 5.0** | **88.7 ± 5.5** | **89.0 ± 3.6** | **97.2 ± 4.1** | F_1,26_  p | 0.08  0.78 | 0.13  0.72 | **4.86**  **0.04** | 0.09 | 0.13 | 0.13 | 0.66 |
| PC C18:0/C18:1 | 100 ± 16.8 | 110.916.6 | 110.08.0 | 120.311.3 | F_1,26_  p | 0.53  0.47 | 0.63  0.43 | 7.65E-04  0.98 | 0.59 | 0.63 | 0.44 | 0.32 |
| PC C18:0/C20:4 | 100 ± 8.4 | 92.0 ± 9.7 | 82.2 ± 8.3 | 95.8 ± 9.3 | F_1,26_  p | 0.69  0.42 | 0.11  0.75 | 1.65  0.21 | 0.13 | 0.51 | 0.26 | 0.72 |
| PE C16:0/C18:1 | 100 ± 4.1 | 90.6 ± 9.3 | 91.1 ± 4.7 | 103.7 ± 6.0 | F_1,26_  p | 0.12  0.73 | 0.07  0.79 | 3.28  0.08 | 0.15 | 0.34 | 0.10 | 0.59 |
| PE C16:0/C20:4 | 100 ± 3.9 | 90.0 ± 7.8 | 99.4 ± 5.1 | 105.2 ± 3.4 | F_1,26_  p | 2.04  0.16 | 0.17  0.68 | 2.37  0.14 | 0.92 | 0.24 | 0.33 | 0.30 |
| PE C18:0/C18:1 | 100 ± 6.0 | 91.19.3 | 87.7 ± 5.1 | 98.1 ± 3.5 | F_1,26_  p | 0.19  0.67 | 0.01  0.90 | 2.47  0.13 | 0.12 | 0.40 | 0.09 | 0.78 |
| PE C18:0/C20:4 | 100 ± 9.3 | 94.4 ± 9.6 | 82.7 ± 9.6 | 98.6 ± 8.0 | F_1,26_  p | 0.58  0.45 | 0.36  0.55 | 1.56  0.22 | 0.19 | 0.66 | 0.19 | 0.91 |
| **PG C16:0/C18:1** | **100 ± 9.7** | **82.8 ± 6.2** | **68.7 ± 10.2** | **92.8 ± 7.5** | F_1,26_  p | 1.80  0.19 | 0.19  0.67 | **6.77**  **0.02** | **0.03** | 0.13 | 0.06 | 0.55 |
| PG C16:0/C20:4 | 100 ± 8.0 | 84.1 ± 12.9 | 79.3 ± 7.3 | 92.7 ± 9.7 | F_1,26_  p | 0.43  0.52 | 0.02  0.89 | 2.53  0.12 | 0.06 | 0.28 | 0.25 | 0.54 |
| **PG C18:0/C18:1** | **100 ± 4.8** | **88.4 ± 10.1** | **74.0 ± 7.8** | **95.5 ± 9.0** | F_1,26_  p | 1.56  0.22 | 0.43  0.52 | **4.73**  **0.04** | **0.01** | 0.29 | 0.08 | 0.63 |
| PG C18:0/C20:4 | 100 ± 7.0 | 83.5 ± 9.3 | 75.1 ± 9.0 | 85.4 ± 8.8 | F_1,26_  p | 2.06  0.16 | 0.15  0.70 | 2.81  0.11 | **0.03** | 0.15 | 0.40 | 0.18 |
| Plasmalogen PC C16:0/20:4 | 100 ± 6.1 | 96.5 ± 8.4 | 94.6 ± 8.5 | 104.4 ± 6.1 | F_1,26_  p | 0.03  0.86 | 0.21  0.65 | 0.93  0.34 | 0.58 | 0.72 | 0.33 | 0.60 |
| Plasmalogen PC C18:0/20:4 | 100 ± 5.0 | 95.3 ± 5.2 | 85.9 ± 7.5 | 93.3 ± 5.4 | F_1,26_  p | 2.25  0.15 | 0.06  0.81 | 1.26  0.27 | 0.11 | 0.50 | 0.41 | 0.35 |
| Plasmalogen PE C16:0/20:4 | 100 ± 7.4 | 80.6 ± 4.0 | 113.9 ± 24.0 | 176.9 ± 61.2 | F_1,26_  p | 3.70  0.07 | 0.58  0.45 | 2.07  0.16 | 0.54 | 0.03 | 0.32 | 0.17 |
| Plasmalogen PE C18:0/20:4 | 100 ± 38.7 | 98.7 ± 53.9 | 87.0 ± 66.8 | 170.4 ± 83.3 | F_1,26_  p | 0.27  0.61 | 0.52  0.48 | 0.56  0.46 | 0.85 | 0.98 | 0.42 | 0.40 |
| Plasmalogen PS C18:0/20:4 | 100 ± 9.1 | 78.4 ± 8.2 | 95.8 ± 13.8 | 87.9 ± 9.7 | F_1,26_  p | 0.08  0.78 | 2.40  0.13 | 0.51  0.48 | 0.79 | 0.08 | 0.62 | 0.35 |
| **PS C16:0/C18:1** | **100 ± 4.8** | **86.0 ± 6.9** | **105.0 ± 7.1** | **108.1 ± 5.2** | **F_1,26_**  **p** | **5.70**  **0.02** | 0.93  0.34 | 2.25  0.15 | 0.53 | 0.10 | 0.72 | 0.24 |
| PS C16:0/C20:4 | 100 ± 5.6 | 86.0 ± 7.9 | 103.1 ± 9.0 | 106.8 ± 6.2 | F_1,26_  p | 3.16  0.09 | 0.58  0.45 | 1.72  0.20 | 0.75 | 0.14 | 0.72 | 0.40 |
| **PS C18:0/C18:1** | **100 ± 8.6** | **81.7 ± 6.7** | **84.3 ± 6.0** | **96.2 ± 6.2** | **F_1,26_**  **p** | 0.01  0.93 | 0.24  0.63 | **5.22**  **0.03** | 0.14 | 0.09 | 0.16 | 0.71 |
| PS C18:0/C20:4 | 100 ± 5.8 | 95.8 ± 4.1 | 93.5 ± 8.0 | 105.7 ± 7.7 | F_1,26_  p | 0.08  0.78 | 0.44  0.51 | 1.88  0.18 | 0.49 | 0.53 | 0.26 | 0.53 |
| Sphinganine | 100± 7.5 | 43.6 ± 11.4 | 89.9 ± 20.4 | 85.4 ± 27.5 | F_1,26_  p | 0.95  0.34 | 3.50  0.07 | 2.54  0.12 | 0.61 | 0.00 | 0.89 | 0.57 |
| Sphingomyelin PC C16:0 | 100 ± 10.2 | 94.8 ± 11.5 | 111.9 ± 6.8 | 113.7 ± 12.1 | F_1,26_  p | 2.48  0.13 | 0.03  0.86 | 0.13  0.72 | 0.33 | 0.72 | 0.89 | 0.37 |
| Sphingomyelin PC C18:0 | 100 ± 6.7 | 97.0 ± 10.1 | 105.2 ± 5.4 | 112.6 ± 8.2 | F_1,26_  p | 1.99  0.17 | 0.09  0.77 | 0.49  0.49 | 0.53 | 0.79 | 0.43 | 0.22 |
| Sphingomyelin PC C18:1 | 100 ± 12.7 | 98.7 ± 12.7 | 117.4 ± 6.4 | 105.1 ± 15.0 | F_1,26_  p | 1.08  0.31 | 0.35  0.56 | 0.23  0.63 | 0.23 | 0.94 | 0.43 | 0.78 |
| **Sphingomyelin PC C20:4** | **100 ± 10.2** | **90.8 ± 17.4** | **115.9 ± 6.4** | **124.1 ± 11.9** | **F_1,26_**  **p** | **4.42**  **0.05** | 1.7E-03  0.97 | 0.55  0.46 | 0.20 | 0.63 | 0.52 | 0.12 |
| Sphingosine | 100 ± 18.5 | 88.2 ± 21.5 | 144.2 ± 17.1 | 112.3 ± 26.0 | F_1,26_  p | 3.06  0.09 | 1.25  0.27 | 0.26  0.61 | 0.08 | 0.66 | 0.29 | 0.68 |
| TAG C16:0/C16:0/C16:0 | 100 ± 22.8 | 66.5 ± 17.0 | 133.2 ± 41.2 | 77.0 ± 12.2 | F_1,26_  p | 0.87  0.36 | 3.67  0.07 | 0.24  0.63 | 0.45 | 0.23 | 0.18 | 0.38 |
| TAG C16:0/C18:1/C16:0 | 100 ± 29.2 | 107.0 ± 20.7 | 125.4 ± 28.6 | 141.7 ± 13.3 | F_1,26_  p | 1.66  0.21 | 0.25  0.62 | 0.04  0.84 | 0.52 | 0.84 | 0.61 | 0.24 |
| TAG C16:0/C20:4/C16:0 | 100 ± 17.4 | 82.0 ± 15.1 | 51.8 ± 11.0 | 80.8 ± 12.8 | F_1,26_  p | 3.26  0.08 | 0.16  0.69 | 2.95  0.10 | **0.03** | 0.42 | 0.09 | 0.37 |
| TAG C18:0/C18:0/C18:0 | 100 ± 25.4 | 79.4 ± 20.7 | 88.6 ± 31.6 | 135.5 ± 23.8 | F_1,26_  p | 0.89  0.35 | 0.31  0.58 | 2.03  0.17 | 0.77 | 0.51 | 0.22 | 0.30 |
| TAG C18:0/C18:1/C18:0 | 100 ± 23.5 | 87.0 ± 31.5 | 84.7 ± 33.1 | 121.8 ± 22.6 | F_1,26_  p | 0.14  0.71 | 0.21  0.65 | 0.91  0.35 | 0.69 | 0.73 | 0.34 | 0.49 |

Targeted metabolomic measurements were performed via multiple-reaction monitoring (MRM) mass spectrometry on lipid extracts from the brains of Ts65Dn and 2N mice treated with vehicle or JZL184. Metabolite levels are shown relative to brains from 2N vehicle-treated mice. Two-way ANOVA F ratios and p values, as well as Student’s T-test p values for are shown. The values reaching statistical significance (p < 0.05) are shown in bold.

Abbreviations:

AcMAGE 2-acetyl monoalkylglycerol ether (MAGE)

acyl carnitine acyl carnitine

acyl DHAP dihydroxyacetone phosphate wtih an ester-linked acyl chain

alkyl glycerone phosphate dihydroxyacetone phosphate with an ether-linked alkyl chain

alkyl LPA lysophosphatidic acid (LPA) with an sn-1 ether-linkage

alkyl LPC lysophophatidylcholine (LPC) with an sn-1 ether linkage

alkyl LPE lysophosphatidylethanolamine (LPE) with an sn-1 ether linkage

alkyl LPG lysophosphatidylglycerol (LPG) with an sn-1 ether linkage

alkyl LPI lysophosphatidyl inositol (LPI) with an sn-1 ether-linkage

alkyl LPS lysophosphatidyl serine (LPS) with an sn-1 ether linkage

alkyl PA phosphatidic acid (PA) with an sn-1 ether linkage

alkyl PC phosphatidylcholine (PC) with an sn-1 ether linkage

alkyl PE phosphatidylethanolamine (PE) with an sn-1 ether linkage

alkyl PG phosphatidylglycerol (PG) with an sn-1 ether linkage

alkyl PI C16:0/18:1 phosphatidyl inositol (PI) with an sn-1 ether linkage

alkyl PS phosphatidyl serine (PS) with an sn-1 ether linkage

DAG diacylglycerol

DHA dihydroxyacetone

EPA eicosapentaenoic acid (C20:5)

FFA C16:0 palmitic acid

FFA C18:0 stearic acid

FFA C18:1 oleic acid

FFA C20:4 arachidonic acid

LPA lysophosphatidic acid (LPA)

LPC lysophosphatidylcholine (LPC)

LPE lysophosphatidylethanolamine (LPE)

LPG lysophosphatidylglycerol (LPG)

LPI lysophosphatidyl inositol (LPI) with an sn-1 ether-linkage

LPS lysophosphatidylserine (LPS)

MAG monoacylglycerol (e.g. 2-AG)

MAGE monoalkylglycerol ether

NAE N-acyl ethanolamine (e.g. anandamide)

PA phosphatidic acid (PA)

PAF/LPC C16:0 2-acetyl lysophosphatidylcholine. a.k.a. platelet activating factor (PAF)

PC phosphatidylcholine (PC)

PE phosphatidylethanolamine (PE)

PG phosphatidylglycerol (PG)

PGD2 prostaglandin D2

PGE2 prostaglandin E2

PGF2alpha prostaglandin F2a

PGJ2 prostaglandin J2

PI phosphatidyl inositol (PI)

Plasmalogen PC phosphatidylcholine (PC) with an sn-1 vinyl ether linkage

Plasmalogen PE phosphatidylethanolamine (PE) with an sn-1 vinyl ether linkage

Plasmalogen PS phosphatidylserine (PS) with an sn-1 vinyl ether linkage

PS phosphatidyl serine (PS)

Sphingomyelin PC sphingomyelin with a PC head-group

TAG triacylglycerol

txb2 thromboxane b2
